# Supplementary material for: Quality of life among adults with scabies: A community-based cross-sectional study in north-western Ethiopia
Source: PLoS Negl Trop Dis. 2024 Aug 20;18(8):e0012429. doi: 10.1371/journal.pntd.0012429 (PMC11364464; doi:10.1371/journal.pntd.0012429)
Supplement: Attachment — Submitted filename: PNTD-D-23-00994_R1_reviewer edited SL.pdf [file pntd.0012429.s002.pdf]

PLOS Neglected Tropical Diseases

Quality of life among adults with scabies: a community-based cross-sectional study in north-western Ethiopia

--Manuscript Draft--

**Manuscript Number:**	PNTD-D-23-00994R1
**Full Title:**	Quality of life among adults with scabies: a community-based cross-sectional study in north-western Ethiopia
**Short Title:**	Quality of life among adults with scabies
**Article Type:**	Research Article
**Keywords:**	Scabies, Dermatologic Life Quality, Ethiopia, Neglected Tropical Disease

Abstract:

Introduction
Introduction
Scabies undermines quality of life through its adverse disease symptoms, by distorting self-perception, and secondary to social stigma. Knowledge of its effect on quality of life in general and on specific aspects of day-to-day life is key to addressing comprehensive health needs of individual patients and to evaluating gains from community-based disease control interventions.
Objectives
To measure the effect of scabies on the quality of life of people with the infestation.
Methods
A community-based cross-sectional study was conducted in a scabies outbreak-affected district in north-western Ethiopia. The study involved 381 households and 86 adults with scabies. We used the ten-item Cardiff Dermatology Life Quality Index (DLQI) tool to collect data. Cronbach's alpha value was used to determine the internal consistency of the Amharic version of the scale. Overall and Dermatology Life Quality (DLQ) domain specific mean scores were calculated. The association between sociodemographic characteristics and scabies-related life quality impairment was tested using Kruskal-Wallis test.
Results
Scabies moderately affected the quality of life of adults with scabies. The overall mean DLQI (mDLQI) score was 9.2 (SD=7.6). 'Symptoms and feelings' and 'daily activity' DLQ domains had the highest mDLQI scores (3.5, SD=1.9 and 2.2, SD=2.5, respectively). 'Leisure activities' was the least affected domain 0.8 (SD=1.1). In terms of severity, scabies had moderate, severe, or very severe effect on DLQ of 54.7% of the participants. However, no association was observed between sociodemographic characteristics and quality of life impairment.
Conclusion
Quality of life was moderately impaired among people affected by scabies. Accompanying scabies treatment with symptom management and health education, against stigma associated with scabies, may help remove this impairment.

**Additional Information:**	
**Question**	**Response**
**Government Employee**<br><br>Are you or any of the contributing authors an employee of the United States government?<br><br>Manuscripts authored by one or more US Government employees are not copyrighted, but are licensed under a CC0 Public Domain Dedication, which allows	No - No authors are employees of the U.S. government.

unlimited distribution and reuse of the article for any lawful purpose. This is a legal requirement for US Government employees.<br><br>This will be typeset if the manuscript is accepted for publication.	
**Financial Disclosure**<br><br>Enter a financial disclosure statement that describes the sources of funding for the work included in this submission. Review the submission guidelines for detailed requirements. View published research articles from *PLOS Neglected Tropical Diseases* for specific examples.<br><br>This statement is required for submission and **will appear in the published article** if the submission is accepted. Please make sure it is accurate.<br><br>**Funded studies**<br>Enter a statement with the following details:<br>• Initials of the authors who received each award<br>• Grant numbers awarded to each author<br>• The full name of each funder<br>• URL of each funder website<br>• Did the sponsors or funders play any role in the study design, data collection and analysis, decision to publish, or preparation of the manuscript?<br><br>Did you receive funding for this work?	Yes
Please add funding details.<br>as follow-up to "**Financial Disclosure**<br><br>Enter a financial disclosure statement that describes the sources of funding for the work included in this submission. Review the submission guidelines for detailed requirements. View published research articles from *PLOS Neglected Tropical Diseases* for specific examples.	GD received the financial support from the National Institute for Health and Care Research (Grant 16/136/29). The views expressed in this article are of the authors, the NHS, the NIHR or the Department of Health and Social Care did not take part in developing the article.

This statement is required for submission and **will appear in the published article** if the submission is accepted. Please make sure it is accurate.<br><br>**Funded studies**<br>Enter a statement with the following details:<br>• Initials of the authors who received each award<br>• Grant numbers awarded to each author<br>• The full name of each funder<br>• URL of each funder website<br>• Did the sponsors or funders play any role in the study design, data collection and analysis, decision to publish, or preparation of the manuscript?<br><br>Did you receive funding for this work?"	
Please select the country of your main research funder (please select carefully as in some cases this is used in fee calculation).<br>as follow-up to "**Financial Disclosure**<br><br>Enter a financial disclosure statement that describes the sources of funding for the work included in this submission. Review the submission guidelines for detailed requirements. View published research articles from *PLOS Neglected Tropical Diseases* for specific examples.<br><br>This statement is required for submission and **will appear in the published article** if the submission is accepted. Please make sure it is accurate.<br><br>**Funded studies**<br>Enter a statement with the following details:<br>• Initials of the authors who received each award<br>• Grant numbers awarded to each author<br>• The full name of each funder<br>• URL of each funder website<br>• Did the sponsors or funders play any role in the study design, data collection and analysis, decision to publish, or preparation of the manuscript?	UNITED KINGDOM - GB

Did you receive funding for this work?"	
**Competing Interests**<br><br>On behalf of all authors, disclose any competing interests that could be perceived to bias this work.<br><br>This statement will be typeset if the manuscript is accepted for publication.<br><br>Review the instructions link below and PLOS NTDs' competing interests policy to determine what information must be disclosed at submission.	There is no conflict of interest between the authors.
**Data Availability**<br><br>Provide a **Data Availability Statement** in the box below. This statement should detail where the data used in this submission can be accessed. This statement will be typeset if the manuscript is accepted for publication.<br><br>Before publication, authors are required to make all data underlying their findings fully available, without restriction. Review our PLOS Data Policy page for detailed information on this policy. Instructions for writing your Data Availability statement can be accessed via the Instructions link below.	Authors will share the data upon request.

Manuscript
Click here to access/download;Manuscript;Dermatologic life quality_PlosNTDs_Mar01_24_Clean.docx

Quality of life among adults with scabies: a community-based cross-sectional study in north-western Ethiopia

Robel Yirgu[1,2,4*], Jo Middleton[1,3], Jackie A Cassell[1,3], Stephen Bremner[1,3], Gail Davey[1,2], Abebaw Fekadu[1,4]

[1]NIHR Global Health Research Unit on Neglected Tropical Diseases, Brighton and Sussex Medical School, Falmer, UK. [2]School of Public Health, Addis Ababa University, Addis Ababa, Ethiopia. [3]Department of Primary Care and Public Health, Brighton and Sussex Medical School, Falmer, UK. [4]Center for Innovative Drug Development and Therapeutic Trials for Africa, Addis Ababa University, Addis Ababa, Ethiopia.

*Correspondence: Robel Yirgu, yirgurob@gmail.com

Abstract

Introduction

Scabies undermines quality of life through its adverse disease symptoms, by distorting self-perception, and secondary to social stigma. Knowledge of its effect on quality of life in general and on specific aspects of day-to-day life is key to addressing comprehensive health needs of individual patients and to evaluating gains from community-based disease control interventions.

Objectives

To measure the effect of scabies on the quality of life of people with the infestation.

Methods

A community-based cross-sectional study was conducted in a scabies outbreak-affected district in north-western Ethiopia. The study involved 381 households and 86 adults with scabies. We used the ten-item Cardiff Dermatology Life Quality Index (DLQI) tool to collect data. Cronbach's alpha value was used to determine the internal consistency of the Amharic version of the scale. Overall and Dermatology Life Quality (DLQ) domain specific mean scores were calculated. The association between sociodemographic characteristics and scabies-related life quality impairment was tested using Kruskal-Wallis test.

Results

Scabies moderately affected the quality of life of adults with scabies. The overall mean DLQI (mDLQI) score was 9.2 (SD=7.6). 'Symptoms and feelings' and 'daily activity' DLQ domains had the highest mDLQI scores (3.5, SD=1.9 and 2.2, SD=2.5, respectively). 'Leisure activities' was the least affected domain 0.8 (SD=1.[illegible]n terms of severity, scabies had moderate, severe, or very severe effect on DLQ of 54.7% of the participants. However, no association was observed between sociodemographic characteristics and quality of life impairment.

Conclusion

Quality of life was moderately impaired among people affected by scabies. Accompanying scabies treatmen[illegible]ith symptom management and health education, against stigma associated with scabies, may help remove this impairment.

Keywords: Scabies, Dermatology Life Quality, Ethiopia, Neglected Tropical Disease

Author summary

Individual's quality of life can be impaired secondary to a range of social, economic, or health related factors. Following the success of biomedical sciences in enhancing life expectancy, improving quality of life has become a crucial health care outcome. Skin diseases undermine quality of life. Disturbing skin sensations such as pain, itch, and irritation from skin lesions and associated social stigma contribute to quality of life impairment. Scabies is a parasitic infestation of the outermost layer of the skin. Disease symptoms such as papular rash, mainly involving the crevices of the body, severe generalized itch and stigma attached to the infestation is believed to impair the quality of life of scabies patients. However, there is limited evidence on the relationship between scabies and quality of life. This study provides rare data on severity of impairment and the most affected aspects of patients' day-to-day life by scabies. Due to the subjective nature of quality of life, more studies are needed in range of settings to contextualize and inform local scabies management protocols.

Introduction

The World Health Organization (WHO) defines quality of life as "an individual's perception of their position in life in the context of the culture and the value systems in which they live and in relation to their goals, expectations, standards, and concerns" (1). This definition is further qualified, in health research, to account for the impact of diseases on health outcomes including physical, mental, and emotional wellbeing (2).

Dermatologic diseases undermine quality of life by altering physical appearance, through social stigma, and secondary to debilitating disease symptoms (3, 4). The severity of their impact is determined by the size of lesions, the affected body parts, and disease chronicity (5, 6).

Scabies is a parasitic skin infestation caused by the mite *Sarcoptes Scabiei* (7). Manifestations of classical scabies include severe itch and papulo-vesicular rash primarily involving crevices of the body such as the axilla, the wrist, the finger webs, the buttocks, and the genitalia in men (7, 8).

Scabies was recently adopted by the WHO as a neglected tropical disease (9). Its high disease burden; its skewed distribution towards under-resourced communities; and amenability to control interventions were among the criteria for the designation. The revitalized attention to scabies capitalized the control efforts of local actors and international organizations (10).

The focus of classic scabies case management is eliminating the mite (7, 11). Manifestations of scabies infestation that undermine the quality of life of patients such as skin irritation and itch do not receive the necessary attention. Lack of evidence on scabies epidemiology and its effect on quality of life and psychosocial wellbeing of patients partly explains the narrowed focus area in scabies case management (12, 13). Data explaining quality of life impairment secondary to scabies is limited (13-15). This study assessed the effect of scabies on quality of life of the affected people using community-based Dermatology Life Quality Index (DLQI) data (16).

Methods

Study design

This population based cross sectional study used the DLQI tool (16) to determine the effect of scabies on quality of life. It was conducted as part of a baseline survey for a larger research project that aimed to measure the off-target effect of ivermectin-based mass drug administration (MDA) for onchocerciasis control on scabies prevalence. The full design of the wider project is detailed elsewhere (17, 18). Here we summarize the aspects of data collection and analysis specifically related to quality of life.

Study area

We conducted the study from 5th to 23rd December 2018 in Ayu Guagusa district, north-western Ethiopia, a region that has been affected by scabies outbreak since 2015 (19). Scabies prevalence at the time of this study was estimated at 13.4% (18). Despite the high prevalence, there were no community-based scabies control interventions in the study district. Providing standard care at primary health care facilities was the only intervention against the outbreak. Other than scabies, Ayu Guagusa district was also endemic to onchocerciasis and targeted by the national onchocerciasis elimination program that entails a bi-annual ivermectin based MDA since 2015. The district comprises 21 predominantly rural *kebeles* (the lowest administrative unit with an average of 500 households) (20) and all 6 study kebeles were rural. Three health centers provide primary health care services to the district population. Health Extension Workers (HEWs), a cadre of community health workers trained for one year, provide disease prevention and health promotion services at health posts (20, 21) and each of the study *kebeles* had one health post.

Source population

All adult residents of Ayu Guagusa district who were diagnosed with scabies

Study population

Adult members of the sample study households with clinically diagnosed scabies.

Eligibility criteria

Adult regular members of the study households or visitors who had stayed with the family for the previous two weeks and who were diagnosed with scabies.

Sampling

Ayu Guagusa district was purposively selected for being the only district in Agew Awi zone where ivermectin-based MDA against onchocerciasis was underway, a criteria relevant to the bigger project (18). From the 21 *kebeles* comprising the district, six (Dekuna Dereb, Arbit, Degera, Ambera, Enavara, and Chibachibasa) were randomly selected using simple random sampling. The *kebeles* were comprised of a varying number of *Gotes* (small villages) and one *Gote* was randomly selected from each study *kebele*. A sampling frame was prepared based on a census of households in the study *Gotes* from which 381 households were selected. All consenting adult members of the sample households that presented with scabies symptoms were examined. The sampling procedure is further explained elsewhere (18).

Data collection procedures

Household heads or adult household members were the informants for household characteristic-related questions. Subsequently, all individual household members, who were available at the time of the survey, were interviewed and examined for manifestations suggestive of scabies. Mid-level health workers from the closest primary hospital to the study area conducted the data collection. Clinical examination, which omitted private body parts, was conducted inside the house of the respective participants, after ensuring privacy.

Sample size

Sample size was calculated using the formula for a continuous outcome variable in single group studies. The sample size (n=71) was adequate to identify quality of life mean score ($\bar{x}$=10) (13) with a standard deviation of (SD=5.9) and margin of error 1.3.

Data collected

The Cardiff DLQI tool was used following the guidance by Finlay and Khan (16). The tool is translated to 55 languages in 20 countries to measure the effect of more than 36 skin conditions, including scabies, on quality of life of patients (16, 22-24). The Amharic translation was validated in 2007 (23).

The DLQI comprises 10 items that measure patients' DLQ over a one-week recall period. Finlay and Khan recommend employing all the ten items in measuring DLQ (16). However, two of the items were excluded in the current study. Item 6 (sports), due to the rarity of organized sporting activities in this rural study community (25). Item number 10 (treatment) for lack of relevance to population surveys where there is no treatment of cases involved (16).

The treatment item was originally deigned to capture the effect of anti-scabietic creams, a source of inconvenience to patients, on quality of life. However, this study was conducted in a community setting and no treatment of patients was involved. Each item had four ordinal response categories with their respective scores (i.e. Not at all=0, a little=1, a lot=2, and very much=3) (16). The highest and lowest total scores using the original index are 30 and 0, respectively, with high scores representing more impaired quality of life. However, in this study the total score using the adapted index was 24.

Clinical examination of the study participants was conducted by five nurses from the local primary hospital. Although all had ample experience caring for scabies patients, a three-day refresher training was given on scabies diagnosis and the DLQI scale by a dermatovenerologist from Bahir Dar University. The team collected sociodemographic data and examined people of all ages who presented with scabies symptoms by going house-to-house; however, for this study we used data only from adults.

Data analysis

The data were analysed using *Stata 14.0* (StataCorp LLC, Texas) statistical software. The ten items are grouped to address six life quality domains that are relevant to dermatological conditions (i.e., symptoms and feelings, daily activities, work and school, leisure, personal relationship, and treatment) (16).

The total and DLQ domain specific mean scores (mDLQI) and standard deviations were calculated. The five domains have a varying maximum possible mean scores. The ‘symptoms and feelings’, ‘daily activity’, and ‘personal relationship’ domains were calculated out of 6 and ‘leisure’ and ‘work and school’ domains out of 3. The difference in ranks of DLQI score by sociodemographic characteristics of participants was determined using Kruskal-Wallis test (26).

The Hongbo *et.al.*, degrees of severity scores were applied to determine the severity of impairment (i.e., no effect (0-1), small effect (2-5), moderate effect (6-10), very severe effect (11-20), and extremely severe effect (21-30)) (27). This grading was developed based on the standard DLQI scale with a maximum score of 30, as opposed to the highest score in the current study, 24. This difference in the two scores was adjusted by prorating the scores from each interval of severity to the standard scale (23). As the amount of missing data was very small, we have simply reported this descriptively.

Ethics

Ethical clearance was received from Addis Ababa University Institutional Review Board (IRB) and the Brighton and Sussex Medical School (BSMS) Research Governance and Ethics Committee (RGEC) with reference numbers AAUMF 03-008 and ER/BSMS9G1Z/1, respectively. Permission was sought from Amhara Regional Health Bureau and local administrative heads before the field work. Written informed consent was obtained from all participants at enrolment. A community-to-facility referral slip was issued at the end of each interview, so that people affected by scabies could get medical care from the nearest health centre.

Results

Study participant profile

Of the approached 91 people identified in 83 (21.7%) households 86 (94.5%) participated in the study. The mean age of participants was 33 and most were between the ages 18 and 30 years. Nearly three quarters of participants had not had formal education, and only 14.1% had secondary (or above) education. Most participants (81.4%) were married. Unmarried, divorced, and widowed participants were grouped as ‘single’ comprising only 18.6% of participants. The main stay of occupation was farming (87.2%) and 4.7% of the participants were unemployed. The distribution of households across wealth quintiles was comparable with a slight preponderance of households categorized under the highest wealth quintile (32.6%) (Table 1).

Table 1. Sociodemographic characteristics of study participants (n=86)

Variables	Categories	n (%)
Sex	Female	41 (47.7%)
	Male	45 (52.3%)
Age	18 – 30	41 (47.7%)
	31 – 40	11 (12.8%)
	>41	34 (39.5%)
	Mean age (SD)	38 (15)
Level of education (n=85)	No formal education	63 (74.1%)
	Primary education	10 (11.8%)
	Secondary or above	12 (14.1%)
Occupation	Farmer	75 (87.2%)
	Student	7 (8.1%)
	Unemployed	4 (4.7%)
Marital status	Married	70 (81.4%)
	Single	16 (18.6%)

Household size	< 5 people	36 (41.9%)
	≥5 people	50 (58.1%)
Household wealth	Lowest	13 (15.1%)
	Second	18 (20.9%)
	Middle	13 (15.1%)
	Fourth	14 (16.3%)
	Highest	28 (32.6%)

Quality of life among people with scabies

The reliability of the Amharic version of the scale was good with Cronbach's α value 0.93 (>0.7). Cumulative mDLQI score among participants was 9.2 (SD=7.6). This corresponds to moderate severity according to Hongbo (27). 'Symptoms' and 'sexual difficulties' items had the highest and lowest proportions of participants reporting extreme quality of life impairment (50.0% and 8.2%, respectively). Slightly more than half of the participants reported no effect against the items 'Work and study', 'clothes', 'daily activities', and 'personal relationship' items (Table 2).

Table 2. Distribution of dermatologic life quality index items among adults with scabies (n=86)

Variable	Categories	Overall n (%)
Over the last week, how itchy, sore, painful, or stinging has your skin been (symptoms)	Very much	43 (50.0%)
	A lot	31 (36.1%)
	A little	7 (8.1%)
	Not at all	5 (5.8%)
Over the last week, how embarrassed or self-conscious have you been because of your skin (feelings)	Very much	29 (33.7%)
	A lot	13 (15.1%)
	A little	7 (8.1%)
	Not at all	37 (43.0%)
Over the last week, how much has your skin interfered with you going shopping or looking after your home or garden (work or study)	Very much	20 (23.3%)
	A lot	14 (16.3%)
	A little	7 (8.1%)
	Not at all	45 (52.3%)
Over the last week, how much has your skin influenced the clothes you wear (clothes)	Very much	24 (27.9%)
	A lot	7 (8.1%)
	A little	4 (4.6%)
	Not at all	51 (59.3%)
Over the last week, how much has your skin affected any social or leisure activities (daily activities) (n=82)	Very much	13 (15.9%)
	A lot	11 (13.4%)
	A little	12 (14.6%)
	Not at all	46 (56.1%)
Over the last week how much has your skin been a problem at work or studying (work or study) (n=85)	Very much	20 (23.5%)
	A lot	10 (11.8%)
	A little	3 (3.5%)
	Not at all	52 (61.2%)
Over the last week, how much has your skin created problems with your partner or any of your close friends or relatives (personal relationships)	Very much	15 (17.4%)
	A lot	10 (11.6%)
	A little	13 (15.1%)
	Not at all	48 (55.8%)
Over the last week, how much has your skin caused any sexual difficulties (sexual difficulties) (n=85)	Very much	7 (8.2%)
	A lot	5 (5.9%)
	A little	8 (9.4%)
	Not at all	65 (76.5%)

We did not observe a statistically significant difference in ranks of the total DLQI score by sociodemographic characteristics of participants or symptom duration. However, married people and people living in households of the lowest wealth quintile reported higher quality of life impairment than their counterparts (Table 3).

Table 3. Demographic characteristics associated with a difference in quality of life of adults with scabies (n=86)

**Variables**	**Categories**	**n (%)**	**Mean ± SD**	**P-value***
Sex	Female	47 (51.7%)	9.2 ± 7.9	0.9
	Male	44 (48.4%)	9.0 ± 7.8	
Age (years)	18 – 30	41 (47.7%)	6.7 ± 7.0	0.4
	31 – 40	11 (12.8%)	8.4 ± 7.7	
	>41	34 (39.5%)	10.5 + 7.9	
Level of education (n=85)	No formal education	63 (74.1%)	8.9 ± 7.8	0.9
	Primary education	10 (11.8%)	9.9 ± 7.6	
	Secondary and above	12 (14.1%)	9.6 ± 8.3	
Occupation	Farmer	75 (87.2%)	9.1 ± 7.7	0.9
	Student	7 (8.1%)	9.3 ± 8.9	
	Unemployed	4 (4.7%)	8.3 ± 7.9	
Marital status	Married	70 (81.4%)	9.4 ± 8.0	0.5
	Single	16 (18.6%)	7.9 ± 6.5	
Symptom duration#	Mean number of days with symptoms	-	51.3 ± 54.8	0.3
Household size	< 5 people	36 (41.9%)	8.5 ± 7.1	0.5
	≥ 5 people	50 (58.1%)	9.6 ± 8.2	
Household wealth	Lowest	13 (15.1%)	12.2 ± 8.0	0.6
	Second	18 (20.9%)	7.4 ± 6.4	
	Middle	13 (15.1%)	9.2 ± 7.4	
	Fourth	14 (16.3%)	8.9 ± 8.7	
	Highest	28 (32.6%)	8.8 ± 8.2	

*Difference in DLQI ranks was determined using Kruskal-Wallis test.

The period between the time of the first symptom and data collection.

The symptoms and feelings domain had the highest mean score ($\bar{x}$=3.7) and leisure the lowest ($\bar{x}$=0.8) (standard deviation 1.9 and 1.1 respectively) (Figure 1).

Scabies had severe and extremely severe effect on the quality of life of 13 (15%) and 23 (27%) participants, respectively. Mild or moderate effect was reported in 43 (50%) scabies cases (Figure 2).

Discussion

Quality of life was moderately impaired among people affected by scabies in north-western Ethiopia. The items: 'symptom', 'feelings', and 'work or study' were the main contributors to the overall DLQ impairment. 'Symptoms and feelings' and 'leisure activity' were the highest and lowest scoring DLQ domains. However, there was no difference in ranks of the DLQI score by sociodemographic characteristics of participants or symptom duration.

In this study, we omitted two items among the ten, which form the standard DLQI scale, for lack of relevance to the study community. This may affect comparability of the current findings with literature. However, we assume the effect is minor as organized sporting activities are uncommon in the study area (25). In a study among podoconiosis patients, in southern Ethiopia, the contribution of impairment to sporting activities to the overall impairment was marginal (24).

Comparable to the current findings, studies in China and Brazil reported moderate impairment to quality of life of scabies patients (13, 14). Factors such as degree of alteration to the skin surface; body parts affected; and the type of lesion are important predictors of severity of impairment in people with dermatologic diseases (4, 6). In classical scabies, the primary lesions are papules with typical distribution (28). Their small-size and distribution, which mainly involves parts of the body that can be covered from other people, can minimize the impact on quality of life. This is also true for other parasitic infestations that commonly cause mild alterations to the skin surface such as tungiasis and pediculosis (29, 30). Conversely, NTDs including podoconiosis, leprosy, and leishmaniasis, which cause severe skin lesions and bodily function impairment, have severe or very severe overall impact on quality of life of patients (31-33). Apart from presentation, disease pattern can play part in determining its effect on quality of life. Scabies had been endemic to north-western Ethiopia and an outbreak was reported in 2015 (19), which was not controlled when this study was conducted. Based on our filed observation, we believe the secular trend and large-scale occurrence had contributed to normalizing the disease. This may partly reduce quality of life impairment by ameliorating the attributes that undermine social functioning.

The 'sexual difficulties' item contributed the least to quality of life impairment total score (13). Sexual difficulties or dysfunction in people affected with dermatologic diseases result from physical impairment or psychosocial factors (34). Distorted self-perception; pain and discomfort from the lesions during intercourse bridge skin diseases and their effect on sexuality (35-37). Classical scabies causes small-sized papular lesions with limited alterations to the skin surface (28). This can minimize its impact on sexual appeal and performance before and during sexual

intercourse. Social norms can also explain the limited impairment to sexuality. In most Ethiopian cultures discussing sex is a taboo (38). It is likely that our study participants may reserve themselves from sharing the change in their sexuality secondary to the infestation.

Of the five DLQ domains investigated in this study, the highest scores arose in the ‘symptoms and feelings’ domain as was in the studies from China and Nepal (14-16). This domain is comprised of items measuring adverse skin sensations caused by the infestation and patients’ affective response to the disease manifestations. It includes itch and irritation caused by the primary lesions, or pain and soreness that are often associated with secondary bacterial infections (39). The intense generalized itch (40) and associated sleep disturbance may also feed into the contribution of this domain to the quality of life total score (41, 42). Optimizing symptom management is vital as getting relief from the debilitating symptoms is an important driver of care seeking for scabies (43).

The second item within the symptoms and feelings DLQ domain captures affective response of scabies patients to the disease symptoms. Skin lesions and related alterations of the skin surface undermine the mental well-being of patients causing low self-esteem, social isolation, and at times depression (14, 44, 45). These responses become more intense when social stigma is attached to the disease or its symptoms (44). Scabies lesions, which involve the hands, and the constant generalized itch, predispose scabies patients to stigma contributing to the negative affective response (46, 47). In the study community, where more than three quarters prefer to avoiding scabies patients (18), fear and shame are the commonest responses to the anticipation of developing the infestation (18). This pervasive stigma can have a toll on the mental health of scabies patients warranting psychosocial interventions alongside clinical care.

‘Daily activity’ was the second most affected domain. It measures the impact on participants’ clothing choices and on work and study. Worth *et.al.* reported a comparable severity of impact where scabies patients living in the slums of northeast Brazil had to change into clothing which covered most parts of their body (14). Trousers, long skirts and tops with long sleeves were preferred as they cover most of the lesions and help to prevent potential stigma (48). The impact on clothing is more pronounced in hot weather where people prefer shorts and tank tops to acclimate (14). The impairment to work and study can be a result of the inconvenience caused by the lesions to physical activity. Sleep disturbance secondary to the itch, which worsens at night, also undermines productivity during daytime (28).

The ‘personal relationship’ domain was the third most affected. From the two items in this domain the ‘relationship with family and friends’ was more impaired. In many communities, scabies is associated with poverty and lack of hygiene (42, 48). Such misconceptions can fuel social stigma against scabies patients. A related literature in this same community reported that 37% of the sample preferred to avoid scabies patients (18). These circumstances can lead to patients limiting social relations in fear of rejection. The other reason for limiting social interactions can be to prevent transmission. Patients who are aware of the increased risk of transmission with increasing physical proximity may isolate themselves to protect people who are close to them (48).

Sociodemographic characteristics and duration of disease symptoms were not associated with quality of life impairment. In another study sex shaped the effect of scabies on the quality of life of patients (14). Females were more affected than males and negative self-perception, caused by alterations to the skin, mediated the impairment (14). Contrary to this, the current study was conducted in a traditional community where dresses of women typically cover most parts of their body. This can limit the severity of impairment to quality of life by minimizing the effect on a range of behaviour including clothing preferences; daily activity; and self-perception.

Longer symptom duration is an important predictor of quality of life impairment in other skin NTDs such as leishmaniasis. It correlates with severity and signify possible complications. In the case of scabies, prolonged symptom duration may not necessarily correlate with severity. Earliest scabies research indicated a decrease in the number of mites with an increase in the duration of the infestation (49). However, this is true only if there is no secondary bacterial infection or progression to crusted scabies.

The overall effect of scabies on the quality of life of the sample adult scabies patients was moderate though a quarter experienced an extremely severe effect. Disease symptoms and self-perception items contributed the most to the overall quality of life impairment. Interventions that aim to improve quality of life, alongside the standard treatment, need to include management of disease symptoms. Alleviating stigma through community-based educational interventions can help reduce the impact on psychosocial wellbeing of patients.

AUTHOR CONTRIBUTIONS

Conceptualization: Jo Middleton, Abebaw Fekadu, Jackie Cassell, Gail Davey.

Analysis: Robel Yirgu, Jo Middleton, Stephen Bremner.

Investigation: Robel Yirgu.

Methodology: Robel Yirgu, Jo Middleton, Abebaw Fekadu, Jackie Cassell, Stephen Bremner, Gail Davey.

Supervision: Jo Middleton, Abebaw Fekadu, Jackie Cassell, Gail Davey.

Writing – original draft: Robel Yirgu.

Writing – review & editing: Robel Yirgu, Jo Middleton, Abebaw Fekadu, Jackie Cassell, Stephen Bremner, Gail Davey.

ACKNOWLEDGMENTS

We would like to thank Gimja Bet hospital staff for conducting clinical diagnosis of scabies cases and data collection. Awi Zone and Ayu Guagusa District Health and administration offices for providing administrative support during field work. We are grateful to the study participants for their time.

FINANCIAL SUPPORT

This project was funded by the National Institute for Health and Care Research (Grant 16/136/29). The funders had no role in the study design, data collection and analysis, decision to publish, or preparation of the manuscript.

CONFLICT OF INTEREST

There is no conflict of interest between the authors.

References

1. The World Health Organization Quality of Life assessment (WHOQOL): position paper from the World Health Organization. Soc Sci Med. 1995;41(10):1403-1409.
2. Mayo NE. Dictionary of Quality of Life and Health Outcomes Measurement. 2015.
3. Chernyshov PV. The Evolution of Quality of Life Assessment and Use in Dermatology. Dermatology. 2019;235(3):167-174.
4. Kowalewska B, Jankowiak B, Cybulski M, Krajewska-Kułak E, Khvorik DF. Effect of Disease Severity on the Quality of Life and Sense of Stigmatization in Psoriatics. Clin Cosmet Investig Dermatol. 2021;14:107-121.
5. He Z, Marrone G, Ou A, Liu H, Ma L, Huang Y, et al. Factors affecting health-related quality of life in patients with skin disease: cross-sectional results from 8,789 patients with 16 skin diseases. Health and Quality of Life Outcomes. 2020;18(1):298.
6. Ludwig MW, Oliveira S, Muller MC, Moraes JF. Quality of life and site of the lesion in dermatological patients. An Bras Dermatol. 2009;84(2):143-150.
7. Chosidow O. Scabies. N Engl J Med. 2006;354(16):1718-1727.
8. Heukelbach J, Feldmeier H. Scabies. The Lancet. 2006;367(9524):1767-1774.
9. World Health Organization. Scabies. Neglected Tropical Diseases 2013. Available. https://www.who.int/news-room/fact-sheets/detail/scabies.
10. World Health Organization. WHO Informal Consultation on a Framework for Scabies Control. Manila; 2019.
11. Scheinfeld N. Controlling Scabies in Institutional Settings A Review of Medications, Treatment Models, and Implementation. Am J Clin Dermatol. 2004;5(1):31-37.
12. Centers for Disease Control and Prevention. Framework for Evaluating Public Health Surveillance Systems for Early Detection of Outbreaks: recommendation from the CDC working group. 2004. Contract No.: RR-5.
13. Jin-gang A, Sheng-xiang X, Sheng-bin X, Jun-min W, Song-mei G, Ying-ying D, et al. Quality of life of patients with scabies. Journal of the European Academy of Dermatology and Venereology. 2010;24:1187-1191.
14. Worth C, Heukelbach J, Fengler G, Walter B, Liesenfeld O, Feldmeier H. Impaired quality of life in adults and children with scabies from an impoverished community in Brazil. Int J Dermatol. 2012;51(3):275-282.

15. Paudel S, Pudasaini P, Adhikari S, Pradhan MB, Shekhar Babu KC. Quality of life in patients with scabies: A cross-sectional study using Dermatology Life Quality Index (DLQI) questionnaire. JEADV Clinical Practice. 2023;2(2):399-403.
16. Finlay AY, Khan GK. Dermatology Life Quality Index (DLQI)--a simple practical measure for routine clinical use. Clin Exp Dermatol. 1994;19(3):210-216.
17. Yirgu R, Middleton J, Fekadu A, Davey G, Bremner S, Jones CI, et al. Secondary impact of ivermectin mass drug administration for onchocerciasis on the prevalence of scabies in northern Ethiopia. BMJ Open. 2023.
18. Yirgu R, Middleton J, Fekadu A, Cassell JA, Tesfaye A, Jones CI, et al. Scabies in the Amhara region of northern Ethiopia: a cross-sectional study of prevalence, determinants, clinical presentation, and community knowledge. BMJ Open. 2023;13(e075038).
19. Enbiale W, Ayalew A. Investigation of a Scabies Outbreak in Drought-Affected Areas in Ethiopia. Trop Med Infect Dis. 2018;3(114):1-9.
20. Assefa Y, Gelaw YA, Hill PS, Taye BW, Van Damme W. Community health extension program of Ethiopia, 2003–2018: successes and challenges toward universal coverage for primary healthcare services. Globalization and Health. 2019;15(1):24.
21. Federal Democratic Republic of Ethiopia Ministry of Health. Health Sector Development Programme IV.2010.
22. Lewis V, Finlay AY. 10 years experience of the Dermatology Life Quality Index (DLQI). J Investig Dermatol Symp Proc. 2004;9(2):169-180.
23. Basra MK, Fenech R, Gatt RM, Salek MS, Finlay AY. The Dermatology Life Quality Index 1994-2007: a comprehensive review of validation data and clinical results. Br J Dermatol. 2008;159(5):997-1035.
24. Henok L, Davey G. Validation of the Dermatology Life Quality Index among patients with podoconiosis in southern Ethiopia. Br J Dermatol. 2008;159(4):903-906.
25. Wolde B, Gaudin B. The institutional organization of Ethiopian athletics Annales d'Éthiopie. 2008;XXIII:471-493.
26. Sthle L, Wold S. Analysis of variance (ANOVA). Chemometrics and Intelligent Laboratory Systems. 1989;6(4):259-272.
27. Hongbo Y, Thomas CL, Harrison MA, Salek MS, Finlay AY. Translating the science of quality of life into practice: What do dermatology life quality index scores mean? J Invest Dermatol. 2005;125(4):659-664.
28. Hardy M, Engelman D, Steer A. Scabies: A clinical update. Aust Fam Physician. 2017;46(5):264-268.

29. Walker SL, Lebas E, Sario VD, Deyasso Z, Doni SN, Marks M, et al. The prevalence and association with healthrelated quality of life of tungiasis and scabies in schoolchildren in southern Ethiopia. PLoS Negl Trop Dis. 2017;11(8):1-11.
30. Ngamcherdtrakul P, Yampratoom R, Siriboriruk J, Annanon N. Quality of Life among Children with Head Lice; Before and After Treatment. Journal of The Department of Medical Services. 2019;44(4):54-61.
31. Enbiale W, Verdonck K, Gebeyehu M, van Griensven J, de Vries HJC. Surgical debulking of podoconiosis nodules and its impact on quality of life in Ethiopia. PLoS Negl Trop Dis. 2021;15(1):1-12.
32. Ahmed N, Naeem A, Zahid B, Tahir M, Bashir U, Kausar S, et al. Effect of Cutaneous Leishmaniasis on Quality of Life of Patients, a Multicentric Study in Tertiary Care Hospitals in Pakistan Using DLQI. International Journal of Clinical and Experimental Medical Sciences. 2021;7(4):103-107.
33. Das NK, De A, Naskar B, Sil A, Das S, Sarda A, et al. A Quality of Life Study of Patients with Leprosy Attending the Dermatology OPD of a Tertiary Care Center of Eastern India. Indian J Dermatol. 2020;65(1):42-46.
34. Narang T, Garima, Singh SM. Psychosexual disorders and dermatologists. Indian Dermatology Online Journal. 2016;7(3).
35. Wojciechowska-Zdrojowy M, Reid A, Szepietowski JC, Wojciechowski A. Analysis of Sexual Problems in Men With Psoriasis. J Sex Marital Ther. 2018;44(8):737-745.
36. Molina-Leyva A, Almodovar-Real A, Carrascosa JC, Molina-Leyva I, Naranjo-Sintes R, Jimenez-Moleon JJ. Distribution pattern of psoriasis, anxiety and depression as possible causes of sexual dysfunction in patients with moderate to severe psoriasis. An Bras Dermatol. 2015;90(3):338-345.
37. Duarte GV, Calmon H, Radel G, de Fátima Paim de Oliveira M. Psoriasis and sexual dysfunction: links, risks, and management challenges. Psoriasis (Auckland, NZ). 2018;8:93-99.
38. Tesso DW, Fantahun MA, Enquselassie F. Parent-young people communication about sexual and reproductive health in E/Wollega zone, West Ethiopia: Implications for interventions. Reproductive Health. 2012;9(1):13.
39. Chandlera DJ, Fuller LC. A Review of Scabies: An Infestation More than Skin Deep. Dermatology. 2019;235:79-90.
40. Walton SF, Currie BJ. Problems in Diagnosing Scabies, a Global Disease in Human and Animal Populations. Clin Microbiol Rev. 2007;20(2):268-279.

41. Dagne H, Dessie A, Destaw B, Yallew WW, Gizaw Z. Prevalence and associated factors of scabies among schoolchildren in Dabat district, northwest Ethiopia, 2018. Environ Health Prev Med. 2019;24(67):1-8.
42. Mitchell E, Bell S, Thean LJ, Sahukhan A, Kama M, Koroivueti A, et al. Community perspectives on scabies, impetigo and mass drug administration in Fiji: A qualitative study. PLoS Negl Trop Dis. 2020;14(12):1-19.
43. Trettin B, Lassen JA, Andersen F, Agerskov H. The journey of having scabies–A qualitative study. Journal of Nursing Education and Practice. 2019;9(2):1-9.
44. Bennis I, Thys S, Filali H, De Brouwere V, Sahibi H, Boelaert M. Psychosocial impact of scars due to cutaneous leishmaniasis on high school students in Errachidia province, Morocco. Infect Dis Poverty. 2017;6(1):1-8.
45. Shiva F, Seifi N, Yaghoubi M, Momenzadeh A, Moradi A, Darchini Maragheh E, et al. Evaluation of Quality of Life, Anxiety and Depression in Children with Cutaneous Leishmaniasis. Int J Pediatr. 2019;7(6):9591-9600.
46. Ahmad I, Humayun Ze, Ahmad M. Pattern of cutaneous leishmaniasis cases among troops and their families in sibi. Pak Armed Forces Med J 2008;58(2):209-212.
47. Pal B, Murti K, Siddiqui NA, Das P, Lal CS, Babu R, et al. Assessment of quality of life in patients with post kalaazar dermal leishmaniasis. Health and Quality of Life Outcomes. 2017;15(1):148.
48. Lopes MJ, Silva ETd, Ca J, Gonçalves A, Amabelia, Rodrigues, et al. Perceptions, attitudes and practices towards scabies in communities on the Bijagós Islands, Guinea-Bissau. Trans R Soc Trop Med Hyg. 2020;114(1):49-56.
49. Mellanby K. The development of symptoms, parasitic infection and immunity in human scabies. Parasitology. 1943;35(4):197-206.

Figure

Click here to access/download;Figure;Figure_1_Mean scores of the five quality of life domains.tif

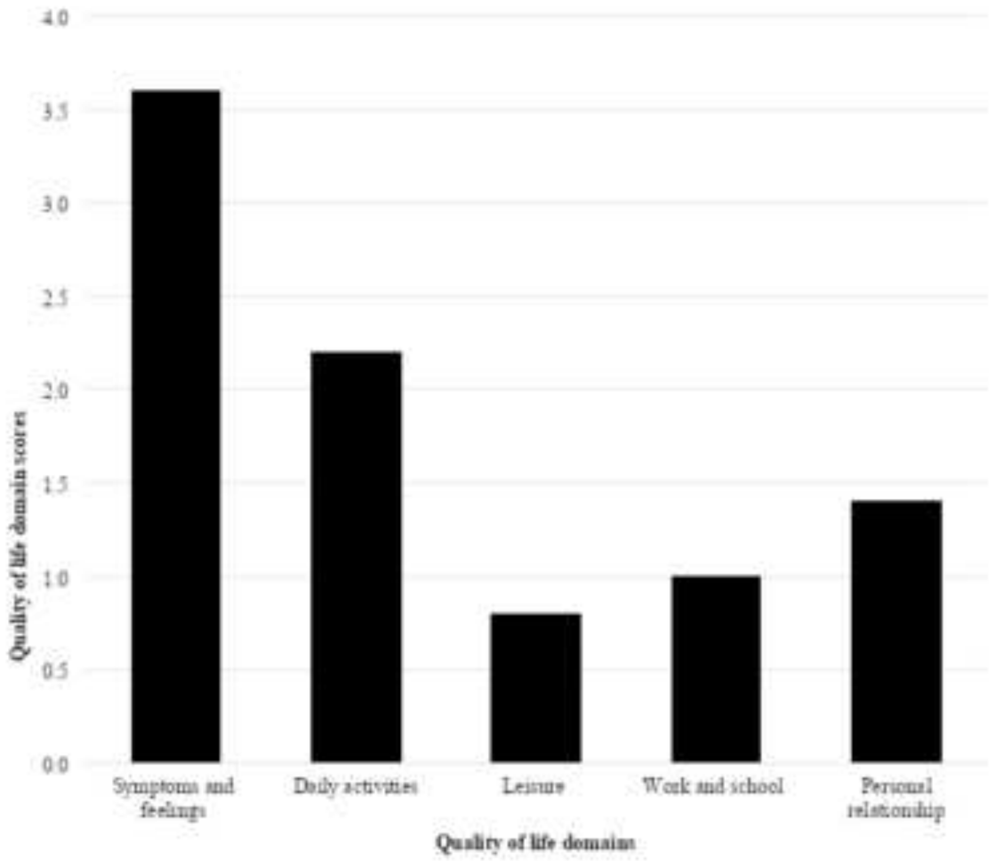

Figure 1. Mean scores of the five quality of life domains (n=86)

Figure

Click here to access/download;Figure;Figure_2_Degrees of severity.tif

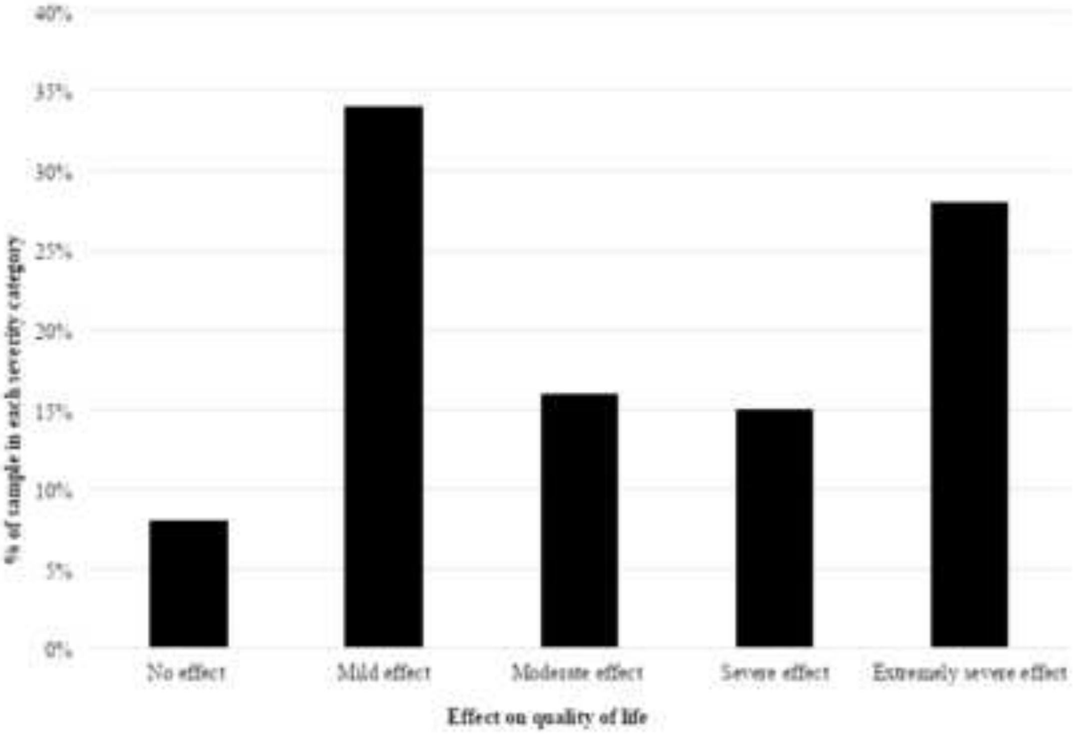

Figure 2. Dermatology quality of life index scores by degrees of severity (n=86)

Point by point response

RE: PNTD-D-23-00994-Quality of life among adults with scabies: a community-based cross-sectional study in north-western Ethiopia

Dear PlosNTDs reviewers and Editors,
Thank you for your continued consideration and the positive review of our manuscript entitled "Quality of life among adults with scabies: a community-based cross-sectional study in north-western Ethiopia".

We have addressed all comments from all the three reviewers. The improvements to the manuscript are indicated in tracked changes. Furthermore, the point by point response, in this document, gathered the changes under each comment for easier visualization.

Thank you again.

Yours,

The manuscript co-authors

	**RESPONSE TO REVIEWERS' COMMENTS**
**1**	**Reviewer#1**
1.1.	**Reviewer**: *The study context is confusing. line "32" outbreak affected ....since 2015, if the study is conducted in a community where there is uncontrolled outbreak .... this by itself has Psychological implication and bias the DLQI measurement. How could the authors explain this?*
	**RESPONSE:** As the reviewer rightly pointed out, descriptions of the study setting benefit from further explanation to set a clear picture of the context to readers. To this end, we have added a few sentences at the manuscript. (see tracked changes manuscript lines 94-100. Inserted text in [square brackets], deleted text ~~strikethrough~~):<br><br>'[Scabies prevalence at the time of this study was estimated at 13.4% (19). Despite the high prevalence, there were no community-based scabies control interventions in the study district. Providing standard care at primary health care facilities was the only

	intervention against the outbreak. Ayu Guagusa was also endemic to onchocerciasis and a bi-annual ivermectin based MDA had been underway since 2015.]’<br><br>**RESPONSE:** We agree that the outbreak may affect the DLQI scores. However, scabies is endemic to the study area and the outbreak is believed to date to before 2015, the year when it was first declared. Though we agree with the reviewer and assume the outbreak may limit the effect of scabies on the quality of life of patients, we could not find evidence to support our claim on the existence and direction of effect. We have included sentences describing this in the manuscript (see tracked changes manuscript lines 344-349):<br><br>‘[Apart from presentation, disease pattern may also play a part in determining its effect on quality of life. Scabies had been endemic to north-western Ethiopia and an outbreak was reported in 2015 (18), which was not controlled when this study was conducted. Based on our field observations, we believe the secular trend and large-scale occurrence had contributed to normalizing the disease. This may partly reduce quality of life impairment by ameliorating the attributes that undermine social functioning.]’
1.2.	**Reviewer**: *Study objectives are clearly stated at the end of the introduction, a clear testable hypothesis is not essential on such a study.*<br>**RESPONSE:** We concur with this comment. The sentence describing the research hypothesis has been removed.
1.3.	**Reviewer**: *In the methods section, the study design is not clearly stated.*<br>**RESPONSE**: We now have included a few words to further clarify the study design (see tracked changes manuscript lines 101-106).<br><br>‘This [prospective population-based] cross sectional study [used the DLQI tool (16) to determine the impact of scabies on quality of life. It was conducted as] part of a baseline survey for a larger research project that measured the off-target effect of ivermectin-based [mass drug administration (]MDA[)] for onchocerciasis control on scabies prevalence. The [full] design of the [wider] project is detailed elsewhere (17). [Here we summarize the aspects of data collection and analysis specifically related to quality of life.]’
1.4.	**Reviewer**: *In the study area section: this study occurred during a scabies outbreak? What percentage of the population was affected?*

	**RESPONSE**: The study was conducted in scabies outbreak-affected north-western Ethiopia. A related study in the same community estimated a 13.4% prevalence. (see tracked changes manuscript lines 112-118).<br><br>'[We conducted the study from 5th to 23rd December 2018 in Ayu Guagusa district, north-western Ethiopia, a region that has been affected by scabies outbreak since 2015 (18). Scabies prevalence at the time of this study was estimated at 13.4% (19).]'
1.5.	**Reviewer:** *Had people already received MDA ivermectin?*<br>**RESPONSE**: Thank you for this question. We agree that giving a clear account of the scabies control interventions, in the study area, allows readers a better understanding. There had been a bi-annual ivermectin based MDA for onchocerciasis elimination since 2015. However, there was no MDA for scabies control in Ayu Guagusa district. We revised the manuscript to include a few more sentences that help clarify the scabies control strategies at the time of the study. (see tracked changes manuscript lines 112-118)<br><br>'[Despite the high prevalence, there were no community-based scabies control interventions in the study district. Providing standard care at primary health care facilities was the only intervention against the outbreak. Ayu Guagusa district was also endemic for onchocerciasis. A bi-annual ivermectin based MDA had been underway, since 2015, aiming at onchocerciasis elimination.]'
1.6.	***Reviewer****: Is this a very rural community?*<br>**RESPONSE**: The district comprises 21 predominantly rural *Kebeles* (villages) and all six study *kebeles* were rural. We have revised this section to clarify the study setting (see tracked changes manuscript line 118)<br><br>'The district comprises 21 predominantly rural *kebeles* (the lowest administrative unit with an average of 500 households) and [and all 6 study kebeles were rural 20]'
1.7.	***Reviewer****: A sampling procedure was done to select households in the study area, but then were all household members examined?*<br>**RESPONSE**: All household members who were available at the time of the survey were examined (see tracked changes manuscript lines 143-148).

	‘[Household heads or adult household members were the informants for household characteristic- related questions. Subsequently, all individual household members who were available at the time of the survey were interviewed and examined for manifestations suggestive of scabies.]’
1.8.	***Reviewer****: The sample size was calculated to test a hypothesis? Reference number 20 in this section refers to clinical studies on not studies related to QoL and not sure how reference number 12 is helping in calculating the sample size. Please revise this section carefully.*<br>**RESPONSE**: The sample size was estimated to obtain an adequate sample to determine the effect of scabies on the quality of life of patients (calculated based on median DLQI score). Reference number 22 details the assumptions for using the formula. The second reference in this section was reference number 13 that is meant to indicate source of the median DLQI score, one of the assumptions used in the sample size calculation (see tracked changes manuscript lines 151-154).<br><br>‘[Sample size was calculated using the formula for a continuous outcome variable in single group studies. The sample size (n=71) was adequate to identify quality of life mean score ($\bar{x}$=10) (13) with a standard deviation of (SD=5.9) and margin of error 1.3]’
1.9.	***Reviewer****: Data collection was done by whom? Who diagnosed the patients with scabies? was there a field case-definition used?*<br>**RESPONSE**: Nurses from a nearby primary hospital conducted the clinical diagnosis and collected sociodemographic data. Clinical diagnosis was used to identify scabies patients. However, no field case definition was used. We made changes to the manuscript to clarify the role of nurses in identifying scabies cases. (see tracked changes manuscript lines 176-178).<br><br>‘[The team collected sociodemographic data and examined people who presented with scabies symptoms by going house-to-house. They examined people of all ages for the parent project, however, in this analysis we used data only from adults.]’
1.10.	***Reviewer****: Was there any missing data? How was this handled?*<br>**RESPONSE**: Thanks for this comment, also made by Reviewer 3. We have revised the manuscript to include details about how missing data was handled (see lines 195-196).

	‘[As the amount of missing data was very small, we have simply reported this descriptively.]’
1.11.	***Reviewer:*** *Ref #23 does not explain that sporting activities are rare in this rural study community!*<br>**RESPONSE**: It is true that the old Ref #23 and the now Ref#25 is an article from a study that validated the DLQI tool for use among podoconiosis patients. In this study impairment to sporting activities contributed the least to the overall effect of podoconiosis on the quality of life of the patients. Our intention was to show that the limited effect on sporting activities resulted from the limited organized sporting activities in rural Ethiopia. We included additional reference to back this claim in the current revised version of the manuscript. (see reference number 27).<br><br>‘[27. Wolde B, Gaudin B. The institutional organization of Ethiopian athletics Annales d'Éthiopie. 2008;XXIII:471-493.]’
1.12.	**Reviewer**: With regards to treatment was ivermectin MDA given to these participants? Is traditional treatment not tried in this community?<br><br>**RESPONSE**: 74.7% of the sample had participated in the last MDA against onchocerciasis, which took place six months before this study. The MDA involved administering ivermectin, a drug that can also cure scabies at the right dose. However, the onchocerciasis MDA involved administering a single dose of ivermectin, which is inadequate to cure scabies. This being the case, we studied all adults who were diagnosed with scabies regardless of their participation in the MDA. However, we did not ask participants whether they used traditional treatment to care for their manifestations. This being the case, the use of traditional treatment is pervasive. Previous studies indicated that nearly 80% of patients used traditional medicine to treat different diseases before seeking care from health facilities. Based on this data we assume a considerable proportion of our study participants did use traditional medicine.
1.13.	**Reviewer**: You need a reference for the adapting the cumulative score and prorating the scores.<br>**RESPONSE**: We have included a reference.

1.14.	***Reviewer**: The authors concluded.... timely holistic care.... a bit farfetched and exaggerated. would be good to be explicit what does holistic care entitle give the moderate DLQ implication of Scabies*<br>**RESPONSE**: Thank you for this important comment. Tailoring the conclusion to specific aspects of symptomatic management and the attributes that contribute to the impairment of psychosocial wellbeing provides a better understanding to readers. We have modified the conclusion along this line (see the revised conclusion lines 50-51).<br><br>'[Quality of life was moderately impaired among people affected by scabies. Accompanying scabies treatment with symptomatic management and community based health education campaigns against stigma associated with scabies may help remove this impairment.]'
1.15.	***Reviewer: 12.** Line 24; Scabies undermines ……….. there were only three studies, and reported moderate impact on DLQ…. …. thus is a bit over toned suggest revision.*<br><br>**RESPONSE**: We now see the overstatement. We have made changes to give a more reasonable tone to the sentence. (see the revisions lines 93-95).<br><br>'[Data explaining the effect of scabies on the quality of life impairment secondary to scabies is limited]'
1.16.	***Reviewer**: Line 70; complications of scabies….. left unattended……. suggest to be explicit which complications and give references.*<br>**RESPONSE**: We have modified the sentence in the manuscript specifying which manifestations are important to patients but are not receiving the necessary attention. (see tracked changes manuscript lines 89-91).<br><br>'[Manifestations of scabies infestation that undermine the quality of life of patients such as skin irritation and itch do not receive the necessary attention]'
1.17.	***Reviewer**: Line 17 to 72; lack of evidence …..narrowed case management. If there were known complications which entailed management modifications then what made it narrow? Would these complications also have stake in quality of life of affected individuals.*

	**RESPONSE**: We agree that the article would benefit from clarifying this claim. There is an overall evidence gap concerning which aspect of the infestation contributes the most to quality of life impairment. Among these, the effect on psychosocial wellbeing and social functioning of patients is the least studied. More knowledge on these aspects can push scabies management beyond providing cure to the infestation. (see tracked changes manuscript lines 88-91).<br><br>'[The focus of classic scabies case management is eliminating the mite (7, 11). Often, manifestations of scabies infestation that undermine quality of life of patients such as skin irritation and itch are left unattended. Lack of evidence on scabies and its effect on the quality of life and psychosocial wellbeing of patients partly explains the narrowed areas of focus in scabies case management (12, 13).]'
1.18.	***Reviewer****: Line 165; just for curiosity "unemployed" from the context of the study population……. Living on subsistence mixed farming what does it imply.*<br><br>**RESPONSE**: The study sample is part of a predominantly rural agrarian community where agriculture is the main economic activity. People referred to as unemployed are those who neither participate in agriculture nor in any other income generating activity.
1.19.	***Reviewer****: Lines 259-266; the references cited indicated that degree of alteration, body part affected, ……. are associated with degree of DLQ ….. but in your study such variables were not considered; thus could it be arguable that you under/over estimated the implication of scabies as important variables were not included in your study.*<br>**RESPONSE**: Not measuring the degree of alteration of the skin surface and mapping the most affected body parts is an important limitation of this study. However, the comparison with diseases that cause severe alteration such as podoconiosis and leishmaniasis was meant to signify that extensive alterations to the skin surface predict a more severe quality of life impairment. In a counter argument the relatively mild scabies lesions caused a moderate impairment. (see tracked changes manuscript lines 332-334)<br><br>'[Comparable to the current findings, studies in China and Brazil reported moderate impairment to quality of life of scabies patients (13, 14). Factors such as degree of alteration to the skin surface; body parts affected; and the type of lesion are important predictors of severity of impairment in people with dermatologic diseases (4, 6).]'

1.20.	***Reviewer:*** *Line 273; sexuality: no reference is given to your claim of "taboo". Also in subsequent paragraphs; from line 299 to 305..... in discussing the implication of scabies in personal relationships …… risky sexual behavior is indicated. In your result it was not seen as such, any reason.*<br><br>**RESPONSE**: A reference is cited to back the community's unfavourable perception of discussing sex.<br><br>'[Tesso DW, Fantahun MA, Enquselassie F. Parent-young people communication about sexual and reproductive health in E/Wollega zone, West Ethiopia: Implications for interventions. Reproductive Health. 2012;9(1):13.]'<br><br>**RESPONSE**: In this study, scabies had a marginal impact on the sexual life of the sample participants. Though anecdotal, the study community perceives scabies more as a disease of poverty and poor hygiene than as a sexually transmitted disease. In the related descriptive study (doi:10.1136/bmjopen-2023-075038.) only 4% of the sample associated scabies with commercial sex workers.
1.21.	***Reviewer:*** *The exclusion of two items from the standard DLQI scale is mentioned, but the rationale for their exclusion is not discussed in detail. Providing more information on why these items were considered irrelevant could add transparency to the study's methodology.*<br><br>**RESPONSE**: We have further elaborated the paragraph to justify the justify the exclusion of the two items from the scale in this study (see tracked changes manuscript lines 161-168).<br><br>'[However, two of the items were excluded in the current study. Item 6 (sports), due to the rarity of organized sporting activities in this rural study community (26). Item number 10 (treatment) for lack of relevance to population surveys where there is no treatment of cases involved (16). The treatment item was originally deigned to capture the effect of anti-scabietic creams, a source of inconvenience to patients, on quality of life. However, this study was conducted in a community setting and no treatment of patients was

	involved. Each item had four ordinal response categories with their respective scores (i.e. Not at all=0, a little=1, a lot=2, and very much=3) (16)]’.
	**Reviewer#2**
2.1.	***Reviewer****: This being a cross-sectional study, have the authors consider completing a STROBE checklist and adding as an appendix?*<br>**RESPONSE**: We looked up and checked all elements of STROBE checklist are addressed in the manuscript.
2.2.	***Reviewer****: In the abstract, best to mention how many participants responded (n=86) rather than how many were approached (91)*<br>**RESPONSE**: Thank you for this comment. Changes have been made to the abstract to report the number of people who participated instead of the number invited to participate (see tracked changes manuscript lines 209-210).<br><br>‘[The study involved 381 households and 86 adults with scabies participated in the study. We used the ten item Cardiff Dermatologic Life Quality Index (DLQI) tool for data collection.]’
2.3.	***Reviewer****: The results match the data analysis plan given. Can the authors describe data handling? How was it collected, entered and analysed using which programme?*<br><br>**RESPONSE**: Thank you for the valid comment. We have elaborated the data collection procedures. This is a community-based study and data collectors went house to house collecting socio-demographic data and conducting clinical examination on adults who presented with scabies symptoms. We have also described the software package used for the analysis (see tracked changes manuscript lines 176-180).<br><br>‘[The team collected sociodemographic data and examined people who presented with scabies symptoms by going house-to-house. They examined people of all ages for the parent project; however, in this analysis we used data only from adults.<br>The data were analysed using *Stata 14.0* (StataCorp LLC, Texas) statistical software.]’
2.4.	***Reviewer****: Line 178 - correct mis-spelling "closes".*<br>**RESPONSE**: Thank you for pointing this out. We have corrected the spelling error.

2.5.	***Reviewer**: The results are clearly presented, but I have some questions: why was age categorization done this way?*<br><br>**RESPONSE**: Existing evidence indicates high scabies burden mong children and adolescents. Categorizing age in short intervals is more informative. However, among adults there is a relatively low prevalence and a uniform distribution across age groups. For this reason, we opted to use data driven categorization.
2.6.	***Reviewer**: lines 204-205 : better state "we did not observe statistically significant difference..." and maybe comments on some of the differences seen; for example participants in smaller households scored higher.*<br><br>**RESPONSE**: Thank you for the comment, we have modified this (see tracked changes manuscript lines 271-274).<br><br>'[We did not observe a statistically significant difference in ranks of the total DLQI score by sociodemographic characteristics of participants or symptom duration. However, married people and people living in households of the lowest wealth quintile reported higher quality of life impairment than their counterparts.]'
2.7.	***Reviewer**: Table 3, the variable "symptom duration" is not described and difficult to understand meaning of result.*<br><br>**RESPONSE**: We agree that this could be unclear to readers. We have included a foot note describing "symptom duration"<br><br>'[# The period between the time of the first symptom and data collection.]
2.8.	***Reviewer**: Fig 1 , comparing mean of QoL domains this way may not be very clear. Some domains are scored out of 6, and others out of 3. The percentage of domain scores could be calculated from the total possible score for each domain and a comparatively more accurate figure obtained.*<br>**RESPONSE**: We share the reviewers' views on this. The domains were calculated based on a varying maximum domain mean score. We agree that changing the mean scores into percentage can contribute to better clarity of the paper. However, the existing cut-off scores to determine degree of severity (Hongbao et.al.,) is calculated based on a

	cumulative score of all the items in the scale. This limits its application to determine severity of impairment per individual domains. Nonetheless, we have revised the manuscript to further explain how each domain specific mean scores was calculated (see tracked changes manuscript lines 185-187).<br><br>'[The five domains have a varying maximum possible mean scores. The 'symptoms and feelings', 'daily activity', and 'personal relationship' domains were calculated out of 6 and 'leisure' and 'work and school' domains out of 3. The difference in ranks of DLQI score by sociodemographic characteristics of participants was determined using Kruskal-Wallis test (26).]'
2.9.	***Reviewer****: Fig 2 , 27% of participants reported that their QoL was extremely severely affected. This important result is not brought out in your conclusion.*<br>**RESPONSE**: We have revised the manuscript so that this finding is well reflected in the conclusion.<br><br>'[The overall effect of scabies on the quality of life of the sample adult scabies patients was moderate and a quarter experienced extremely severe effect. Disease symptoms and self-perception items contributed the most to the overall quality of life impairment. Interventions that aim to improve quality of life, alongside the standard treatment, need to include symptom management.]'
2.10.	***Reviewer****: Can the authors comment on how many of the participants diagnosed with scabies and interviewed came from the same household in which others were also affected?*<br><br>**RESPONSE**: All scabies cases came from 83 (21.7%) households. From these households 21% contributed 2 or more people affected with scabies to the total sample.
2.11.	***Reviewer****: This section needs careful revision to relate/ compare the results of this study to findings in other scabies QoL study and QOL in other dermatological conditions in Ethiopia for each paragraph. For example, paragraph starting at line 290 on daily activities: how is this related to the present study participants?*<br><br>**RESPONSE:** This section has undergone significant revisions and included literature to enhance comparisons with other NTDs.

2.12.	***Reviewer**: One important limitation was that the severity of scabies in participant was not recorded or the body parts affected (on line 287 - there is a comments about hands...)*<br><br>**RESPONSE**: Existing evidence shows that the hands are one of the most commonly affected parts of the body with scabies lesions. Though in our study we didn't map body parts affected by the lesion, we assume the bodily distribution of lesions indicated in the literature holds true. For further reference:<br>https://link.springer.com/chapter/10.1007/978-3-031-26070-4_15
2.13.	***Reviewer**: Line 307 - "male sex is a strong predictor of QoL" - this statement needs clarification*<br>**RESPONSE**: Thank you for taking note of and pointing out this inconsistency with previous publications. It was a typographical error that we have corrected. We have also added a few more details on the association between sex and quality of life impairment (see tracked changes manuscript lines 408-412).<br><br>'[A study in Brazil reported that sex plays an important role in shaping the effect of scabies on the quality of life of patients (14). Females were more affected than males and negative self-perception caused by alterations to the skin mediated the impairment (14).]'
2.14.	***Reviewer**: The conclusions on lines 311-313 do not seem to be linked to your findings: self -perception and social functioning were the least affected QoL scores in your findings. Which finding reflect the stigmatizing effect of scabies in this group of participants?*<br><br>**RESPONSE**: This conclusion is based on the effect of scabies self-perception and social functioning. This is shown in item number 2 in Table 2 and the daily activity domain in figure 1.
	**Reviewer#3**
3.1.	***Reviewer**: The data analysis section is concise, but it would be beneficial to include more details on how missing data, if any, was handled and whether any statistical software was used. Additionally, specifying the statistical tests used for comparisons would enhance the transparency of the analysis.*

	**RESPONSE**: We fully agree with this comment. We have revised the manuscript to include details about how missing data were handled (see lines 195-196).<br><br>'[As the amount of missing data was very small, we have simply reported this descriptively.]'<br><br>**RESPONSE**: We now have described the statistical software (see line 159) and statistical tests used in comparing the data (see lines 180-181).<br><br>'[The data were analysed using *Stata 14.0* (StataCorp LLC, Texas) statistical software.]'<br><br>'[The total and DLQ domain specific mean scores (mDLQI) and standard deviations were calculated. The difference in ranks of DLQI score by sociodemographic characteristics of participants was determined using the Kruskal-Wallis test (27).]'
3.2.	***Reviewer****: The discussion could benefit from a more explicit comparison with similar studies conducted in other regions or countries. This would provide a broader context for understanding whether the impact on quality of life in northern Ethiopia is consistent with or differs from findings in diverse cultural and geographical settings.*<br><br>**RESPONSE**: We agree with the reviewer. However, literature on the effect of scabies on quality of life of patients is limited. In an extensive literature search, we could only find two articles on the subject and both are referenced in the discussion. This has limited our ability to compare our findings with other studies. Nonetheless, following this comment by the reviewer we have tried to expand the discussion by making comparisons with literature on other NTDs in Ethiopia.
3.3.	***Reviewer****: The discussion mentions that sociodemographic characteristics were not associated with quality of life impairment. However, it could be valuable to elaborate on why certain sociodemographic factors were expected to have an impact and explore potential reasons for the lack of association in this specific context.*<br><br>**RESPONSE**: The lack of association between sociodemographic characteristics and quality of life impairment is further elaborated in line with your comments (please see line 408-415).

	'[Sociodemographic characteristics and duration of disease symptoms were not associated with quality of life impairment. In another study sex shaped the effect of scabies on the quality of life of patients (14). Females were more affected than males and negative self-perception, caused by alterations to the skin, mediated the impairment (14). Contrary to this, the current study was conducted in a traditional community where dresses of women typically cover most parts of their body. This can limit the severity of impairment to quality life by minimizing the effect on a range of behaviour including clothing preferences; daily activity; and self-perception.]'
3.4.	***Reviewer****: The discussion mentions stigma and social isolation as significant contributors to quality of life impairment. Providing more in-depth insights into the nature and extent of stigma, as well as potential interventions to address it, would contribute to a more nuanced understanding.*<br><br>**RESPONSE**: This is a valid comment. The scope of stigma and the necessary interventions to avert its impact on the well-being of scabies patients is further explained in the revised manuscript (see tracked changes manuscript lines 379-383 & 393-402).<br><br>'[In the study community, where more than three quarters prefer to avoiding scabies patients (18), fear and shame are the commonest responses to the anticipation of developing the infestation (18). This pervasive stigma can have a toll on the mental health of scabies patients warranting psychosocial interventions alongside clinical care.]'<br><br>'[In many communities, scabies is associated with poverty and lack of hygiene (44, 50). Such misconceptions can fuel social stigma against scabies patients. A related literature in this same community reported that 37% of the sample preferred to avoid scabies patients (18). These circumstances can lead to patients limiting social relations in fear of rejection. The other reason for limiting social interactions can be to prevent transmission. Patients who are aware of the increased risk of transmission with increasing physical proximity may isolate themselves to protect people who are close to them (50).]'
3.5.	***Reviewer****: While the sampling procedure is mentioned, it lacks a detailed explanation of how the random selection of households was conducted. Providing a step-by-step*

	description or referencing the specific publication where it is explained would be beneficial for transparency and reproducibility.<br>**RESPONSE**: Thank you for this input. We have included a reference, from the larger project, that details the sampling procedure step-by-step (See reference number 17 in the bibliography).<br><br>'[Yirgu R, Middleton J, Fekadu A, Cassell JA, Tesfaye A, Jones CI, et al. Scabies in the Amhara region of northern Ethiopia: a cross-sectional study of prevalence, determinants, clinical presentation, and community knowledge. BMJ Open. 2023;13(e075038).]'

Click here to access/download
Revised Article with Changes Highlighted
Dermatologic life quality_PlosNTDs_Mar01_24_Tracked.docx
