# Supplementary material for: Quality of life among adults with scabies: A community-based cross-sectional study in north-western Ethiopia
Source: PLoS Negl Trop Dis. 2024 Aug 20;18(8):e0012429. doi: 10.1371/journal.pntd.0012429 (PMC11364464; doi:10.1371/journal.pntd.0012429)
Supplement: Attachment — Submitted filename: PNTD-D-23-00994_R2_reviewer.pdf [file pntd.0012429.s004.pdf]

PLOS Neglected Tropical Diseases

Quality of life among adults with scabies: a community-based cross-sectional study in north-western Ethiopia

--Manuscript Draft--

**Manuscript Number:**	PNTD-D-23-00994R2
**Full Title:**	Quality of life among adults with scabies: a community-based cross-sectional study in north-western Ethiopia
**Short Title:**	Scabies infestation and quality of life
**Article Type:**	Research Article
**Keywords:**	Scabies, Dermatologic Quality of Life, Ethiopia, Neglected Tropical Disease
**Abstract:**	Introduction<br>Scabies undermines quality of life through its highly disturbing disease symptoms, by distorting self-perception, and secondary to social stigma. Knowledge of its effect on quality of life in general and on specific aspects of day-to-day life is key to addressing comprehensive health needs of individual patients and to evaluating gains from community-based disease control interventions.<br>Objectives<br>To measure the effect of scabies on the quality of life of people with the infestation.<br>Methods<br>A community-based cross-sectional study was conducted in a scabies outbreak-affected district in north-western Ethiopia. The study involved 381 households and 86 adults with scabies. We used the ten-item Cardiff Dermatology Life Quality Index (DLQI) tool to collect data. Cronbach's alpha value was used to determine the internal consistency of the Amharic version of the scale. Overall and Dermatology Life Quality (DLQ) domain specific mean scores were calculated. The association between sociodemographic characteristics and scabies-related life quality impairment was tested using Kruskal-Wallis test.<br>Results<br>Scabies moderately affected the quality of life of adults with scabies. The overall mean DLQI (mDLQI) score was 9.2 (SD=7.6). 'Symptoms and feelings' and 'daily activity' DLQ domains had the highest mDLQI scores (3.5, SD=1.9 and 2.2, SD=2.5, respectively). 'Leisure activities' was the least affected domain 0.8 (SD=1.1). In terms of severity, scabies had moderate orsevere effect on DLQ of 54.7% of the participants extremely severe effect was reported among 27% of participants. However, no association was observed between sociodemographic characteristics and quality of life impairment.<br>Conclusion<br>Quality of life was moderately impaired among people affected by scabies. Refocusing attention on management of disease symptoms, under the standard scabies treatment, and providing psychosocial support to improve self-perception of people affected with scabies may help reduce quality of life impairment.
**Additional Information:**	
**Question**	**Response**
**Government Employee**<br><br>Are you or any of the contributing authors an employee of the United States government?<br><br>Manuscripts authored by one or more US Government employees are not copyrighted, but are licensed under a CC0	No - No authors are employees of the U.S. government.

Powered by Editorial Manager® and ProduXion Manager® from Aries Systems Corporation

Public Domain Dedication, which allows unlimited distribution and reuse of the article for any lawful purpose. This is a legal requirement for US Government employees.<br><br>This will be typeset if the manuscript is accepted for publication.	
**Financial Disclosure**<br><br>Enter a financial disclosure statement that describes the sources of funding for the work included in this submission. Review the submission guidelines for detailed requirements. View published research articles from *PLOS Neglected Tropical Diseases* for specific examples.<br><br>This statement is required for submission and **will appear in the published article** if the submission is accepted. Please make sure it is accurate.<br><br>**Funded studies**<br>Enter a statement with the following details:<br>• Initials of the authors who received each award<br>• Grant numbers awarded to each author<br>• The full name of each funder<br>• URL of each funder website<br>• Did the sponsors or funders play any role in the study design, data collection and analysis, decision to publish, or preparation of the manuscript?<br><br>Did you receive funding for this work?	Yes
Please add funding details.<br>as follow-up to "**Financial Disclosure**<br><br>Enter a financial disclosure statement that describes the sources of funding for the work included in this submission. Review the submission guidelines for detailed requirements. View published research articles from *PLOS Neglected Tropical Diseases* for specific examples.	GD received the financial support from the National Institute for Health and Care Research (Grant 16/136/29). The views expressed in this article are of the authors, the NHS, the NIHR or the Department of Health and Social Care did not take part in developing the article.

This statement is required for submission and **will appear in the published article** if the submission is accepted. Please make sure it is accurate.<br><br>**Funded studies**<br>Enter a statement with the following details:<br>• Initials of the authors who received each award<br>• Grant numbers awarded to each author<br>• The full name of each funder<br>• URL of each funder website<br>• Did the sponsors or funders play any role in the study design, data collection and analysis, decision to publish, or preparation of the manuscript?<br><br>Did you receive funding for this work?"	
Please select the country of your main research funder (please select carefully as in some cases this is used in fee calculation).<br>as follow-up to "**Financial Disclosure**<br><br>Enter a financial disclosure statement that describes the sources of funding for the work included in this submission. Review the submission guidelines for detailed requirements. View published research articles from *PLOS Neglected Tropical Diseases* for specific examples.<br><br>This statement is required for submission and **will appear in the published article** if the submission is accepted. Please make sure it is accurate.	UNITED KINGDOM - GB

Powered by Editorial Manager® and ProduXion Manager® from Aries Systems Corporation

**Funded studies**<br>Enter a statement with the following details:<br>• Initials of the authors who received each award<br>• Grant numbers awarded to each author<br>• The full name of each funder<br>• URL of each funder website<br>• Did the sponsors or funders play any role in the study design, data collection and analysis, decision to publish, or preparation of the manuscript?<br><br>Did you receive funding for this work?"	
**Competing Interests**<br><br>On behalf of all authors, disclose any competing interests that could be perceived to bias this work.<br><br>This statement will be typeset if the manuscript is accepted for publication.<br><br>Review the instructions link below and PLOS NTDs' competing interests policy to determine what information must be disclosed at submission.	There is no conflict of interest between the authors.
**Data Availability**<br><br>Provide a **Data Availability Statement** in the box below. This statement should detail where the data used in this submission can be accessed. This statement will be typeset if the manuscript is accepted for publication.<br><br>Before publication, authors are required to make all data underlying their findings fully available, without restriction. Review our PLOS Data Policy page for detailed information on this policy. Instructions for writing your Data Availability statement can be accessed via the Instructions link below.	Authors will share the data upon request.

Powered by Editorial Manager® and ProduXion Manager® from Aries Systems Corporation

Manuscript Click here to access/download;Manuscript;Dermatologic life quality_PlosNTDs_May07_24_Clean.docx

Quality of life among adults with scabies: a community-based cross-sectional study in north-western Ethiopia

Robel Yirgu[1,2,4*], Jo Middleton[1,3], Jackie A Cassell[1,3], Stephen Bremner[1,3], Gail Davey[1,2], Abebaw Fekadu[1,4]

[1]NIHR Global Health Research Unit on Neglected Tropical Diseases, Brighton and Sussex Medical School, Falmer, UK. [2]School of Public Health, Addis Ababa University, Addis Ababa, Ethiopia. [3]Department of Primary Care and Public Health, Brighton and Sussex Medical School, Falmer, UK. [4]Center for Innovative Drug Development and Therapeutic Trials for Africa, Addis Ababa University, Addis Ababa, Ethiopia.

*Correspondence: Robel Yirgu, yirgurob@gmail.com

Abstract

Introduction

Scabies undermines quality of life through its highly disturbing disease symptoms, by distorting self-perception, and secondary to social stigma. Knowledge of its effect on quality of life in general and on specific aspects of day-to-day life is key to addressing comprehensive health needs of individual patients and to evaluating gains from community-based disease control interventions.

Objectives

To measure the effect of scabies on the quality of life of people with the infestation.

Methods

A community-based cross-sectional study was conducted in a scabies outbreak-affected district in north-western Ethiopia. The study involved 381 households and 86 adults with scabies. We used the ten-item Cardiff Dermatology Life Quality Index (DLQI) tool to collect data. Cronbach's alpha value was used to determine the internal consistency of the Amharic version of the scale. Overall and Dermatology Life Quality (DLQ) domain specific mean scores were calculated. The association between sociodemographic characteristics and scabies-related life quality impairment was tested using Kruskal-Wallis test.

Results

Scabies moderately affected the quality of life of adults with scabies. The overall mean DLQI (mDLQI) score was 9.2 (SD=7.6). 'Symptoms and feelings' and 'daily activity' ~~DLQ~~ domains had the highest ~~mDLQI~~ scores (3.5, SD=1.9 and 2.2, SD=2.5, respectively). 'Leisure activities' was the least affected domain 0.8 (SD=1.1). In terms of severity, scabies had moderate or severe effect on DLQ of 54.7% of the participants and extremely severe effect was reported among 27% of participants. However, no association was observed between sociodemographic characteristics and quality of life impairment.

Conclusion

Quality of life was moderately impaired among people affected by scabies. Refocusing attention on management of disease symptoms, under the standard scabies treatment, and providing psychosocial support to improve self-perception of people affected with scabies may help reduce quality of life impairment.

Keywords: Scabies, Dermatology Life Quality, Ethiopia, Neglected Tropical Disease

Author summary

Scabies is a parasitic infestation of the outermost layer of the skin. Disease symptoms such as papular rash, mainly involving the crevices of the body, severe generalized itch and stigma ~~attached~~ to the infestation is believed to impair the quality of life of scabies patients. However, there is limited evidence about the relationship between scabies and quality of life. This study presents novel data on quality of life impairment associated with scabies and which aspects of patients' day-to-day lives are the most affected. This study was conducted in scabies outbreak-affected north-western Ethiopia. Scabies had a moderate impact on the quality of life of the affected people. Disease symptoms and their psychosocial impact contributed the most to quality of life impairment. Participants of this study came from different sociodemographic backgrounds. Nonetheless, there were no major differences in DLQI scores by background characteristics.

Introduction

The World Health Organization (WHO) defines quality of life as “an individual’s perception of their position in life in the context of the culture and the value systems in which they live and in relation to their goals, expectations, standards, and concerns” (1). This definition is further qualified, in health research, to account for the impact of diseases on health outcomes including physical, mental, and emotional wellbeing (2).

Dermatologic diseases have a serious impact on the quality of life of those affected. Bodily dissatisfaction, anxiety, and depression have been commonly reported among people affected with skin diseases (3, 4). Given the chronicity of most dermatologic conditions, the physical and psychosocial impacts are often prolonged (5). Psychosocial impacts result from altered physical appearance, social stigma, and debilitating disease symptoms. Severity of impact is determined by the size of lesions, the affected body parts, and symptom duration (5, 6). In previous research sociodemographic characteristics such as sex, age, income, and marital status were associated with quality of life impairment (5-7).

Scabies is a parasitic skin infestation caused by the mite *Sarcoptes Scabiei* (8). Manifestations of classical scabies include severe itch and papulo-vesicular rash primarily involving crevices of the body such as the axilla, the wrist, the finger webs, the buttocks, and the genitalia in men (8, 9).

Scabies was recently adopted by the WHO as a neglected tropical disease (10). Its high disease burden; skewed distribution towards under-resourced communities; and amenability to control interventions were among the criteria for the designation. The revitalized attention to scabies capitalized the control efforts of local actors and international organizations (11).

The focus of classic scabies case management is eliminating the mite (8, 12). Manifestations of scabies infestation that undermine the quality of life of patients such as skin irritation and itch do not receive the necessary attention. Lack of evidence on scabies epidemiology and its effect on quality of life and psychosocial wellbeing of patients partly explains the narrowed focus area in scabies case management (13, 14). Data explaining quality of life impairment secondary to scabies is limited (14-16). This study aimed to determine the overall and most affected quality of life domains among people with scabies by using community-based Dermatologic Life Quality Index (DLQI) data (17). The findings will inform a more comprehensive scabies case management and community based control interventions.

Methods

Study design

We conducted a population-based cross-sectional study. A survey design was chosen considering the descriptive nature of the main objective, which was to determine the effect of scabies on the quality of life of patients. The study was part of a baseline survey for a larger project that aimed to measure the off-target effect of ivermectin-based mass drug administration (MDA) for onchocerciasis elimination on scabies prevalence. The full design of the parent project is detailed elsewhere (18, 19). This article summarized aspects of the data collection and analysis that pertain to quality of life by following the STROBE reporting outline.

Study area

We conducted the study from December 5 to 23, 2018 in Ayu Guagusa district, north-western Ethiopia, a region that had been affected by scabies outbreak since 2015 (20). The prevalence at the time of this study was estimated at 13.4% (19). Despite this high prevalence, there were no community-based scabies control interventions in the study district. Providing standard care at primary health care facilities was the only intervention against the outbreak. However, a bi-annual ivermectin based MDA was underway for onchocerciasis elimination since 2015. The district was comprised of 21 predominantly rural *kebeles* (the lowest administrative unit with an average of 500 households) (21) and all 6 study kebeles were rural. Three health centers provided primary health care services to the district population. Health Extension Workers (HEWs), a cadre of community health workers trained for one year, provide disease prevention and health promotion services at health posts (21, 22) and *kebeles* had one health post each.

Source population

All adult residents of Ayu Guagusa district who were diagnosed with scabies. Children who were screened positive for scabies, in the bigger project, were referred to the nearby primary health care facilities for treatment.

Study population

Adult members of the sample study households with clinically diagnosed scabies.

Eligibility criteria

Adult regular members of the study households and visitors who had stayed with the family for the previous two weeks before the survey and who were diagnosed with scabies.

Sampling

Ayu Guagusa district was purposively selected for being the only district in Agew Awi zone where ivermectin-based MDA against onchocerciasis was underway, a criteria relevant to the bigger project (19). From the 21 *kebeles* comprising the district, six (Dekuna Dereb, Arbit, Degera, Ambera, Enavara, and Chibachibasa) were randomly selected using simple random sampling. The *kebeles* were comprised of a varying number of *Gotes* (small villages) and one *Gote* was randomly selected from each study *kebele*. A sampling frame was prepared based on a census of households in the study *Gotes* from which 381 households were selected. All consenting adult members of the sample households that presented with scabies symptoms were examined. The sampling procedure is further explained elsewhere (19).

Data collection procedures

Household heads or adult household members were the informants for household characteristic-related questions. Subsequently, all individual household members, who were available at the time of the survey, were interviewed and examined for manifestations suggestive of scabies. Five nurses, with ample experience caring for scabies cases, were recruited from the nearby primary hospital to collect data. A dermatovenerologist (WE) from Bahir Dar University gave them a three day refresher training before data collection. The training was meant to standardize scabies diagnosis; introduce the data collection tool; and create awareness about human subject research ethics.

Clinical diagnosis was employed to identify scabies cases. Physical examination of the study participants was conducted inside their house where there was optimal illumination of the examination room. After ensuring privacy, participants were asked to expose body parts that are typically affected by scabietic lesions such as the arms, legs, and the abdomen. However, private body parts were not involved in the examination. Other than physical examination, participants were also asked about contact history with a person presenting scabies manifestations.

Sample size

Sample size was calculated using the formula for a continuous outcome variable in single group studies. The sample size (n=71) was adequate to identify quality of life mean score ($\bar{x}$=10) (14) with a standard deviation of (SD=5.9) and margin of error 1.3.

Data collected

The Cardiff DLQI tool was used following the guidance by Finlay and Khan (17). The tool is translated to 55 languages in 20 countries to measure the effect of more than 36 skin conditions, including scabies, on quality of life of patients (17, 23-25). The Amharic translation was validated in 2007 (23).

The DLQI comprises 10 items that measure patients' DLQ over a one-week recall period. Finlay and Khan recommend employing all the ten items in measuring DLQ (17). However, two of the items were excluded in the current study. Item 6 (sports), due to the rarity of organized sporting activities in this rural study community (26). Item number 10 (treatment) for lack of relevance to population surveys where there is no treatment of cases involved (17). The treatment item was originally designed to capture the effect of anti-scabietic creams, which was not convenient to apply and at times causes skin irritation, on quality of life. ~~However, this~~ study was conducted in a community setting and no treatment of patients was involved. Each item had four ordinal response categories with their respective scores (i.e. Not at all=0, a little=1, a lot=2, and very much=3) (17). The highest and lowest total scores using the original index were 30 and 0, respectively, with high scores representing more impaired quality of life. ~~However, in this~~ study the total score using the adapted index was 24.

Data analysis

The data were analysed using *Stata 14.0* (StataCorp LLC, Texas) ~~statistical software~~. The ~~ten~~ items were grouped to address six life quality domains that are relevant to dermatological conditions (i.e., symptoms and feelings, daily activities, work and school, leisure, personal relationship, ~~and treatment)~~ (17).

The total and DLQ domain specific mean scores (mDLQI) and standard deviations were calculated. The five domains have a varying maximum possible mean scores. The 'symptoms and feelings', 'daily activity', and 'personal relationship' domains were calculated out of 6 and 'leisure' and 'work and school' domains out of 3. The difference in ranks of DLQI score by sociodemographic characteristics of participants was determined using Kruskal-Wallis test (27).

The Hongbo *et.al.*, degrees of severity scores were applied to determine severity of impairment (i.e., no effect (0-1), small effect (2-5), moderate effect (6-10), very severe effect (11-20), and extremely severe effect (21-30)) (28). This grading was developed based on ~~the standard DLQI scale with~~ a maximum score of ~~30~~, ~~as opposed to the highest score in the current study, 24.~~ This

difference in the two scores was adjusted by prorating the scores from each interval of severity to the standard scale (24). The amount of missing data was very small, which we have simply reported.

Ethics

Ethical clearance was received from Addis Ababa University Institutional Review Board (IRB) and the Brighton and Sussex Medical School (BSMS) Research Governance and Ethics Committee (RGEC) with reference numbers AAUMF 03-008 and ER/BSMS9G1Z/1, respectively. Permission was sought from Amhara Regional Health Bureau and local administrative heads before the field work. Written informed consent was obtained from all participants at enrolment. A community-to-facility referral slip was issued at the end of each interview, so that people affected by scabies could get medical care from the nearest health centre.

Results

Study participant profile

Eighty three households (21.7% of screened households) were found to have one or more people affected by scabies. Out of the 91 adults diagnosed with scabies, 86 (94.5%) participated in this study. Data on 5 participants was dropped due to poor quality. The mean age of participants was 33 and most were between the ages 18 and 30 years. Nearly three quarters of participants had not had formal education~~, and only 14.1% had secondary (or above) education~~. Most participants (81.4%) were married. ~~Unmarried, divorced, and widowed participants were grouped as ‘single’ comprising only 18.6% of the participants.~~ The main stay of occupation was farming (87.2%) ~~and 4.7% of the participants were not taking part in any income generating activities.~~ The distribution of households across wealth quintiles was comparable with a slight preponderance of households categorized under the highest wealth quintile (32.6%) (Table 1).

Table 1. Sociodemographic characteristics of study participants (n=86)

Variables	Categories	n (%)
Sex	Female	41 (47.7%)
	Male	45 (52.3%)
Age	18 – 30	41 (47.7%)
	31 – 40	11 (12.8%)
	>41	34 (39.5%)
	Mean age (SD)	38 (15)
Level of education (n=85)	No formal education	63 (74.1%)
	Primary education	10 (11.8%)
	Secondary or above	12 (14.1%)
Occupation	Farmer	75 (87.2%)
	Student	7 (8.1%)
	Unemployed	4 (4.7%)
Marital status	Married	70 (81.4%)
	Single	16 (18.6%)
Household size	< 5 people	36 (41.9%)
	≥5 people	50 (58.1%)
Household wealth	Lowest	13 (15.1%)
	Second	18 (20.9%)
	Middle	13 (15.1%)
	Fourth	14 (16.3%)
	Highest	28 (32.6%)

Quality of life among people with scabies

The reliability of the Amharic version of the scale was good with Cronbach's α value 0.93 (>0.7). Cumulative mDLQI score among participants was 9.2 (SD=7.6). This corresponds to moderate severity according to Hongbo (28). 'Symptoms' and 'sexual difficulties' items had the highest and lowest proportions of participants reporting extreme quality of life impairment (50.0% and 8.2%, respectively). Slightly more than half of the participants reported no effect against the items 'Work and study', 'clothes', 'daily activities', and 'personal relationship' items (Table 2).

Table 2. Distribution of dermatologic life quality index items among adults with scabies (n=86)

Variable	Categories	Overall n (%)
Over the last week, how itchy, sore, painful, or stinging has your skin been (symptoms)	Very much	43 (50.0%)
	A lot	31 (36.1%)
	A little	7 (8.1%)
	Not at all	5 (5.8%)
Over the last week, how embarrassed or self-conscious have you been because of your skin (feelings)	Very much	29 (33.7%)
	A lot	13 (15.1%)
	A little	7 (8.1%)
	Not at all	37 (43.0%)
Over the last week, how much has your skin interfered with you going shopping or looking after your home or garden (work or study)	Very much	20 (23.3%)
	A lot	14 (16.3%)
	A little	7 (8.1%)
	Not at all	45 (52.3%)
Over the last week, how much has your skin influenced the clothes you wear (clothes)	Very much	24 (27.9%)
	A lot	7 (8.1%)
	A little	4 (4.6%)
	Not at all	51 (59.3%)
Over the last week, how much has your skin affected any social or leisure activities (daily activities) (n=82)	Very much	13 (15.9%)
	A lot	11 (13.4%)
	A little	12 (14.6%)
	Not at all	46 (56.1%)
Over the last week how much has your skin been a problem at work or studying (work or study) (n=85)	Very much	20 (23.5%)
	A lot	10 (11.8%)
	A little	3 (3.5%)
	Not at all	52 (61.2%)
Over the last week, how much has your skin created problems with your partner or any of your close friends or relatives (personal relationships)	Very much	15 (17.4%)
	A lot	10 (11.6%)
	A little	13 (15.1%)
	Not at all	48 (55.8%)
Over the last week, how much has your skin caused any sexual difficulties (sexual difficulties) (n=85)	Very much	7 (8.2%)
	A lot	5 (5.9%)
	A little	8 (9.4%)
	Not at all	65 (76.5%)

We did not observe a statistically significant difference in ranks of the total DLQI score by sociodemographic characteristics of participants or mean number of days with scabies symptoms (Table 3).

Table 3. Demographic characteristics associated with a difference in quality of life of adults with scabies (n=86)

Variables	Categories	n (%)	DLQI score mean ± SD	P-value*
Sex	Female	47 (51.7%)	9.2 ± 7.9	0.9
	Male	44 (48.4%)	9.0 ± 7.8	
Age (years)	18 – 30	41 (47.7%)	6.7 ± 7.0	0.4
	31 – 40	11 (12.8%)	8.4 ± 7.7	
	>41	34 (39.5%)	10.5 + 7.9	
Level of education (n=85)	No formal education	63 (74.1%)	8.9 ± 7.8	0.9
	Primary education	10 (11.8%)	9.9 ± 7.6	
	Secondary and above	12 (14.1%)	9.6 ± 8.3	
Occupation	Farmer	75 (87.2%)	9.1 ± 7.7	0.9
	Student	7 (8.1%)	9.3 ± 8.9	
	Unemployed	4 (4.7%)	8.3 ± 7.9	
Marital status	Married	70 (81.4%)	9.4 ± 8.0	0.5
	Single	16 (18.6%)	7.9 ± 6.5	
Household size	< 5 people	36 (41.9%)	8.5 ± 7.1	0.5
	≥ 5 people	50 (58.1%)	9.6 ± 8.2	
Household wealth	Lowest	13 (15.1%)	12.2 ± 8.0	0.6
	Second	18 (20.9%)	7.4 ± 6.4	
	Middle	13 (15.1%)	9.2 ± 7.4	
	Fourth	14 (16.3%)	8.9 ± 8.7	
	Highest	28 (32.6%)	8.8 ± 8.2	

*Difference in DLQI ranks was determined using Kruskal-Wallis test.

The period between the time of the first symptom and data collection.

The symptoms and feelings domain had the highest mean DLQI score 3.7 ± 1.9 and leisure the lowest 0.8 ± 1.1 (Figure 1).

Scabies had severe and extremely severe effect on the quality of life of 13 (15%) and 23 (27%) participants, respectively. Mild or moderate effect was reported in 43 (50%) scabies cases (Figure 2).

Discussion

~~Quality of life was moderately impaired among people affected by scabies in north-western Ethiopia.~~ The items: ‘symptom’, ‘feelings’, and ‘work or study’ were the main contributors to the overall ~~DLQ~~ impairment. ‘Symptoms and feelings’ and ‘leisure activity’ were the highest and lowest scoring DLQ domains. However, there was no difference in DLQI score by sociodemographic characteristics of participants or symptom duration.

In this study, we omitted the sporting activities and treatment items, from the ten that formed the standard DLQI scale, for lack of relevance to the study community. This may affect comparability of the current findings with literature. However, we assume the effect is minor as organized sporting activities were uncommon in the study area (26). In a study among podoconiosis patients, in southern Ethiopia, the contribution of ~~impaired~~ sporting activities to overall impairment was marginal (25).

Comparable to the current findings, studies in China and Brazil reported moderate ~~impairment to quality of life of scabies patients~~ (14, 15). Factors such as degree of alteration to the skin surface; body parts affected; and the type of lesion are important predictors of severity of impairment in people with dermatologic diseases (6, 7). In classical scabies, the primary lesions are papules with typical distribution (29). Their small-size and distribution, which mainly involves parts of the body that can be covered from other people, may minimize the impact on selected life quality domains. This is also true for other parasitic infestations that commonly cause mild alterations to the skin surface such as tungiasis and pediculosis (30, 31). Conversely, other skin NTDs including podoconiosis, leprosy, and leishmaniasis, which cause severe skin lesions and bodily function impairment have severe or very severe overall impact on quality of life of patients (32-34). Apart from presentation, disease pattern can play part in determining its effect on quality of life. Scabies had been endemic to north-western Ethiopia and an outbreak was reported in 2015 (20), which was not controlled at the time of this study. Based on our ~~filed~~ observation, we believe the secular trend and large-scale occurrence had contributed to a progressive reduction in the stigma attached to scabies. This may partly reduce quality of life impairment by ameliorating the attributes that undermine social functioning.

The ‘sexual difficulties’ item contributed the least to quality of life impairment total score (14). Sexual difficulties or dysfunction in people affected with dermatologic diseases result from physical impairment or psychosocial factors (35). Pain and discomfort from the skin lesions and distorted self-perception bridge skin diseases and their effect on sexual function (36-38). Classical

scabies causes small-sized papular lesions with limited alterations to the skin surface (29). This can minimize its impact on sexual appeal and performance before and during sexual intercourse. Social norms can also explain the limited impairment to sexuality. In most Ethiopian cultures discussing sex is a taboo, more so among married couples (39). In our study, more than 80% of the participants were married. It is likely that most were hesitant to discuss any change in sexual function with their partners.

Of the five DLQI domains, investigated in this study, the highest scores arose in the 'symptoms and feelings' domain as was in the studies from China and Nepal (15-17). This domain is comprised of items measuring adverse skin sensations caused by the infestation and patients' affective response to the disease manifestations. It includes itch and irritation caused by the primary lesions, or pain and soreness that are often associated with secondary bacterial infections (40). The intense generalized itch (41) and associated sleep disturbance may also feed into the contribution of this domain to the quality of life total score (42, 43). Optimizing symptom management is vital as getting relief from the debilitating symptoms is an important driver of care seeking for scabies (44).

The second item within the symptoms and feelings DLQ domain captures affective response of scabies patients to the disease symptoms. Skin lesions and related alterations of the skin surface undermine the mental well-being of patients causing low self-esteem, social isolation, and at times depression (15, 45, 46). These responses become more intense when social stigma is attached to the disease or its symptoms (45). Scabies lesions, which involve the hands, and the constant generalized itch, predispose scabies patients to stigma from others contributing to the negative affective response (47, 48). A study among scabies free participants of the same community reported that more than three quarters of participants prefer to avoid scabies patients (19). Fear and shame were the commonest responses to the anticipation of developing the infestation (19). This pervasive stigma can have a toll on the mental health of scabies patients warranting psychosocial interventions alongside clinical care.

'Daily activity' was the second most affected domain. It measures the impact on participants' clothing choices and on work and study. Worth *et.al*. reported a comparable severity of impact where scabies patients living in the slums of northeast Brazil had to change into clothing which covered most parts of their body (15). Trousers, long skirts and tops with long sleeves were preferred as they cover most of the lesions and help to prevent potential stigma (49). The impact on clothing is more pronounced in hot weather where people prefer summer dresses to acclimate

(15). The study population is accustomed to modest clothing with sleeves covering most parts of the body. The impairment to work and study can be a result of the inconvenience caused by the lesions to physical activity. Sleep disturbance secondary to the itch, which worsens at night, also undermines productivity during daytime (29).

The 'personal relationship' domain was the third most affected. From the two items in this domain the 'relationship with family and friends' was more impaired. In many communities, scabies is associated with poverty and lack of hygiene (43, 49). Such misconceptions can fuel social stigma against scabies patients. A related literature in this same community reported that 37% of the sample preferred to avoid scabies patients (19). These circumstances can lead to patients limiting social relations in fear of rejection. The other reason for limiting social interactions can be to prevent transmission. Patients who are aware of the increased risk of transmission with increasing physical proximity may isolate themselves to protect people who are close to them (49). The sociocultural context forms a critical background to the dynamic relationship between the disease and its effect on quality of life. For this reason, future qualitative studies that can delve deeper into sociocultural factors should be conducted to understand the role of community perception and social norms in mediating the impact of scabies on quality of life of patients.

Sociodemographic characteristics and duration of disease symptoms were not associated with quality of life impairment. In another study gender shaped the effect of scabies on the quality of life of patients (15). Females were more affected than males and negative self-perception, caused by alterations to the skin, contributed to the impairment (15). The traditional clothing in our study population, which covers most parts of the body, may limit the severity of impairment by minimizing the effect on clothing preferences; daily activity; and stigma from others. Though we did not find a statistically significant association, the link between sociodemographic characteristics and quality of life impairment among people with scabies needs further research. We suggest multicentre studies with larger sample sizes to investigate potential differences between the different demographic groups with more accuracy.

Longer symptom duration is an important predictor of quality of life impairment in other skin NTDs such as leishmaniasis. It correlates with severity and signify possible complications. In the case of scabies, prolonged symptom duration may not necessarily correlate with severity. Earliest scabies research indicated a decrease in the number of mites with an increase in the duration of the infestation (50). However, this is true only if there is no secondary bacterial infection or progression to crusted scabies.

The overall effect of scabies on the quality of life of the sample adult study participants was moderate though a quarter experienced an extremely severe effect. Disease symptoms and self-perception items contributed the most to the overall quality of life impairment. Interventions that aim to improve quality of life, alongside the standard treatment, need to include management of disease symptoms. Alleviating stigma through community-based educational interventions can help reduce the impact on psychosocial wellbeing of patients.

AUTHOR CONTRIBUTIONS

Conceptualization: Jo Middleton, Abebaw Fekadu, Jackie Cassell, Gail Davey.

Analysis: Robel Yirgu, Jo Middleton, Stephen Bremner.

Investigation: Robel Yirgu.

Methodology: Robel Yirgu, Jo Middleton, Abebaw Fekadu, Jackie Cassell, Stephen Bremner, Gail Davey.

Supervision: Jo Middleton, Abebaw Fekadu, Jackie Cassell, Gail Davey.

Writing – original draft: Robel Yirgu.

Writing – review & editing: Robel Yirgu, Jo Middleton, Abebaw Fekadu, Jackie Cassell, Stephen Bremner, Gail Davey.

ACKNOWLEDGMENTS

We would like to thank Gimja Bet hospital staff for conducting clinical diagnosis of scabies cases and data collection. Awi Zone and Ayu Guagusa District Health and administration offices for providing administrative support during field work. We are grateful to the study participants for their time.

FINANCIAL SUPPORT

This project was funded by the National Institute for Health and Care Research (Grant 16/136/29). The funders had no role in the study design, data collection and analysis, decision to publish, or preparation of the manuscript.

CONFLICT OF INTEREST

There is no conflict of interest between the authors.

References

1. World Health Organization Quality of Life assessment: position paper. Soc Sci Med. 1995;41(10):1403-1409.
2. Mayo NE. Dictionary of Quality of Life and Health Outcomes Measurement. 2015.
3. Yanik M, Gurel MS, Simsek Z, Kati M. The psychological impact of cutaneous leishmaniasis. Clin Exp Dermatol. 2004;29(5):464-467.
4. Kassi M, Kassi M, Afghan AK, Rehman R, Kasi PM. Marring Leishmaniasis: The Stigmatization and the Impact of Cutaneous Leishmaniasis in Pakistan and Afghanistan. PLoS Negl Trop Dis. 2008;2(10):e259.
5. He Z, Marrone G, Ou A, Liu H, Ma L, Huang Y, et al. Factors affecting health-related quality of life in patients with skin disease: cross-sectional results from 8,789 patients with 16 skin diseases. Health and Quality of Life Outcomes. 2020;18(1):298.
6. Ludwig MW, Oliveira Mda S, Muller MC, Moraes JF. Quality of life and site of the lesion in dermatological patients. An Bras Dermatol. 2009;84(2):143-150.
7. Kowalewska B, Jankowiak B, Cybulski M, Krajewska-Kułak E, Khvorik DF. Effect of Disease Severity on the Quality of Life and Sense of Stigmatization in Psoriatics. Clin Cosmet Investig Dermatol. 2021;14:107-121.
8. Chosidow O. Scabies. N Engl J Med. 2006;354(16):1718-1727.
9. Heukelbach J, Feldmeier H. Scabies. The Lancet. 2006;367(9524):1767-1774.
10. World Health Organization. Scabies. Neglected Tropical Diseases 2013. Available: http://www.who. int/neglected_diseases/diseases/scabies/en/.
11. World Health Organization. WHO Informal Consultation on a Framework for Scabies Control. Manila; 2019.
12. Scheinfeld N. Controlling Scabies in Institutional Settings A Review of Medications, Treatment Models, and Implementation. Am J Clin Dermatol. 2004;5(1):31-37.
13. Prevention CfDCa. Framework for Evaluating Public Health Surveillance Systems for Early Detection of Outbreaks;recommendation from the CDC working group. 2004. Contract No.: RR-5.
14. Jin-gang A, Sheng-xiang X, Sheng-bin X, Jun-min W, Song-mei G, Ying-ying D, et al. Quality of life of patients with scabies. Journal of the European Academy of Dermatology and Venereology. 2010;24:1187-1191.
15. Worth C, Heukelbach J, Fengler G, Walter B, Liesenfeld O, Feldmeier H. Impaired quality of life in adults and children with scabies from an impoverished community in Brazil. Int J Dermatol. 2012;51(3):275-282.
16. Paudel S, Pudasaini P, Adhikari S, Pradhan MB, Shekhar Babu KC. Quality of life in patients with scabies: A cross-sectional study using Dermatology Life Quality Index (DLQI) questionnaire. JEADV Clinical Practice. 2023;2(2):399-403.

17. Finlay AY, Khan GK. Dermatology Life Quality Index (DLQI)--a simple practical measure for routine clinical use. Clin Exp Dermatol. 1994;19(3):210-216.
18. Yirgu R, Middleton J, Fekadu A, Davey G, Bremner S, Jones CI, et al. Secondary impact of ivermectin mass drug administration for onchocerciasis on the prevalence of scabies in northern Ethiopia. BMJ Open. 2023.
19. Yirgu R, Middleton J, Fekadu A, Cassell JA, Tesfaye A, Jones CI, et al. Scabies in the Amhara region of northern Ethiopia: a cross-sectional study of prevalence, determinants, clinical presentation, and community knowledge. BMJ Open. 2023;13(e075038).
20. Enbiale W, Ayalew A. Investigation of a Scabies Outbreak in Drought-Affected Areas in Ethiopia. Trop Med Infect Dis. 2018;3(114):1-9.
21. Assefa Y, Gelaw YA, Hill PS, Taye BW, Van Damme W. Community health extension program of Ethiopia, 2003–2018: successes and challenges toward universal coverage for primary healthcare services. Globalization and Health. 2019;15(1):24.
22. Federal Democratic Republic of Ethiopia Ministry of Health. Health Sector Development Programme IV. 2010.
23. Lewis V, Finlay AY. 10 years experience of the Dermatology Life Quality Index (DLQI). J Investig Dermatol Symp Proc. 2004;9(2):169-180.
24. Basra MK, Fenech R, Gatt RM, Salek MS, Finlay AY. The Dermatology Life Quality Index 1994-2007: a comprehensive review of validation data and clinical results. Br J Dermatol. 2008;159(5):997-1035.
25. Henok L, Davey G. Validation of the Dermatology Life Quality Index among patients with podoconiosis in southern Ethiopia. Br J Dermatol. 2008;159(4):903-906.
26. Wolde B, Gaudin B. The institutional organization of Ethiopian athletics Annales d'Éthiopie. 2008;XXIII:471-493.
27. St»hle L, Wold S. Analysis of variance (ANOVA). Chemometrics and Intelligent Laboratory Systems. 1989;6(4):259-272.
28. Hongbo Y, Thomas CL, Harrison MA, Salek MS, Finlay AY. Translating the science of quality of life into practice: What do dermatology life quality index scores mean? J Invest Dermatol. 2005;125(4):659-664.
29. Hardy M, Engelman D, Steer A. Scabies: A clinical update. Aust Fam Physician. 2017;46(5):264-268.
30. Walker SL, Lebas E, Sario VD, Deyasso Z, Doni SN, Marks M, et al. The prevalence and association with healthrelated quality of life of tungiasis and scabies in schoolchildren in southern Ethiopia. PLoS Negl Trop Dis. 2017;11(8):1-11.
31. Ngamcherdtrakul P, Yampratoom R, Siriboriruk J, Annanon N. Quality of Life among Children with Head Lice; Before and After Treatment. Journal of The Department of Medical Services. 2019;44(4):54-61.

32. Enbiale W, Verdonck K, Gebeyehu M, van Griensven J, de Vries HJC. Surgical debulking of podoconiosis nodules and its impact on quality of life in Ethiopia. PLoS Negl Trop Dis. 2021;15(1):1-12.
33. Ahmed N, Naeem A, Zahid B, Tahir M, Bashir U, Kausar S, et al. Effect of Cutaneous Leishmaniasis on Quality of Life of Patients, a Multicentric Study in Tertiary Care Hospitals in Pakistan Using DLQI. International Journal of Clinical and Experimental Medical Sciences. 2021;7(4):103-107.
34. Das NK, De A, Naskar B, Sil A, Das S, Sarda A, et al. A Quality of Life Study of Patients with Leprosy Attending the Dermatology OPD of a Tertiary Care Center of Eastern India. Indian J Dermatol. 2020;65(1):42-46.
35. Narang T, Garima, Singh SM. Psychosexual disorders and dermatologists. Indian Dermatology Online Journal. 2016;7(3).
36. Wojciechowska-Zdrojowy M, Reid A, Szepietowski JC, Wojciechowski A. Analysis of Sexual Problems in Men With Psoriasis. J Sex Marital Ther. 2018;44(8):737-745.
37. Molina-Leyva A, Almodovar-Real A, Carrascosa JC, Molina-Leyva I, Naranjo-Sintes R, Jimenez-Moleon JJ. Distribution pattern of psoriasis, anxiety and depression as possible causes of sexual dysfunction in patients with moderate to severe psoriasis. An Bras Dermatol. 2015;90(3):338-345.
38. Duarte GV, Calmon H, Radel G, de Fátima Paim de Oliveira M. Psoriasis and sexual dysfunction: links, risks, and management challenges. Psoriasis (Auckland, NZ). 2018;8:93-99.
39. Tesso DW, Fantahun MA, Enquselassie F. Parent-young people communication about sexual and reproductive health in E/Wollega zone, West Ethiopia: Implications for interventions. Reproductive Health. 2012;9(1):13.
40. Chandlera DJ, Fuller LC. A Review of Scabies: An Infestation More than Skin Deep. Dermatology. 2019;235:79-90.
41. Walton SF, Currie BJ. Problems in Diagnosing Scabies, a Global Disease in Human and Animal Populations. Clin Microbiol Rev. 2007;20(2):268-279.
42. Dagne H, Dessie A, Destaw B, Yallew WW, Gizaw Z. Prevalence and associated factors of scabies among schoolchildren in Dabat district, northwest Ethiopia, 2018. Environ Health Prev Med. 2019;24(67):1-8.
43. Mitchell E, Bell S, Thean LJ, Sahukhan A, Kama M, Koroivueti A, et al. Community perspectives on scabies, impetigo and mass drug administration in Fiji: A qualitative study. PLoS Negl Trop Dis. 2020;14(12):1-19.
44. Trettin B, Lassen JA, Andersen F, Agerskov H. The journey of having scabies–A qualitative study. Journal of Nursing Education and Practice. 2019;9(2):1-9.
45. Bennis I, Thys S, Filali H, De Brouwere V, Sahibi H, Boelaert M. Psychosocial impact of scars due to cutaneous leishmaniasis on high school students in Errachidia province, Morocco. Infect Dis Poverty. 2017;6(1):1-8.

46. Shiva F, Seifi N, Yaghoubi M, Momenzadeh A, Moradi A, Darchini Maragheh E, et al. Evaluation of Quality of Life, Anxiety and Depression in Children with Cutaneous Leishmaniasis. Int J Pediatr. 2019;7(6):9591-9600.
47. Ahmad I, Humayun Ze, Ahmad M. Pattern of cutaneous leishmaniasis cases among troops and their families in sibi. Pak Armed Forces Med J 2008;58(2):209-212.
48. Pal B, Murti K, Siddiqui NA, Das P, Lal CS, Babu R, et al. Assessment of quality of life in patients with post kalaazar dermal leishmaniasis. Health and Quality of Life Outcomes. 2017;15(1):148.
49. Lopes MJ, Silva ETd, Ca J, Gonçalves A, Amabelia, Rodrigues, et al. Perceptions, attitudes and practices towards scabies in communities on the Bijagós Islands, Guinea-Bissau. Trans R Soc Trop Med Hyg. 2020;114(1):49-56.
50. Mellanby K. The development of symptoms, parasitic infection and immunity in human scabies. Parasitology. 1943;35(4):197-206.

Click here to access/download;Figure;Figure_1_Mean scores of the five quality of life domains.tif

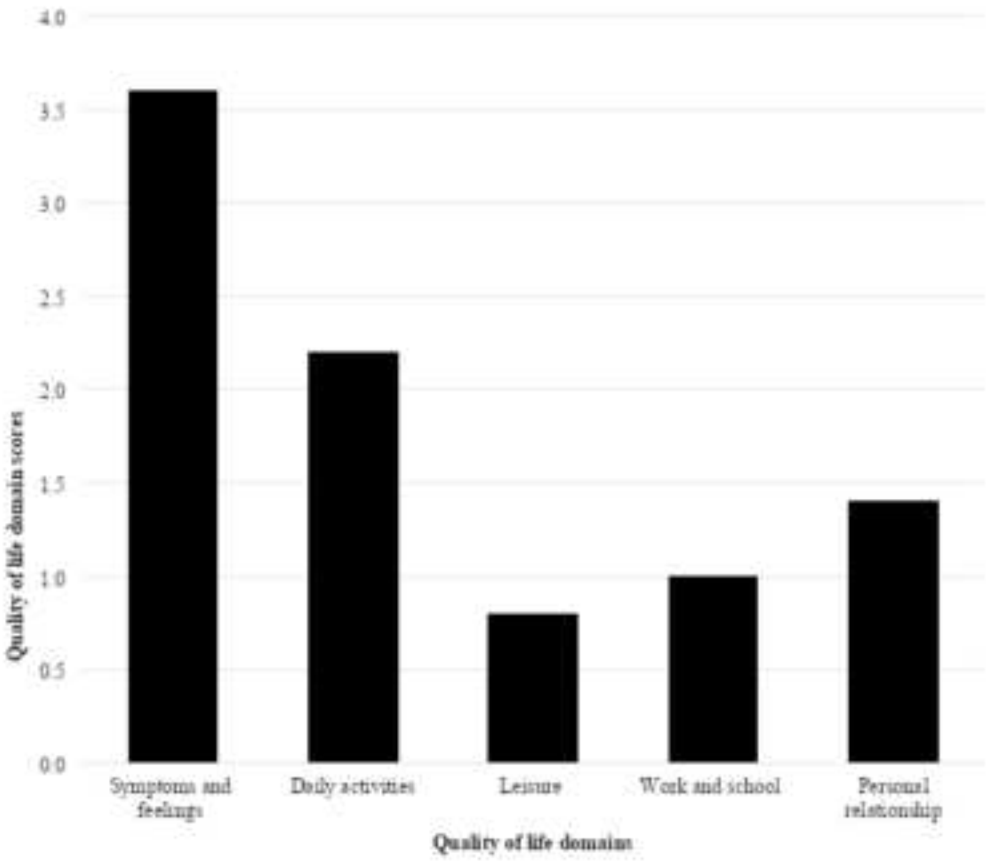

Figure 1. Mean scores of the five quality of life domains (n=86)

Click here to access/download;Figure;Figure_2_Degrees of severity.tif

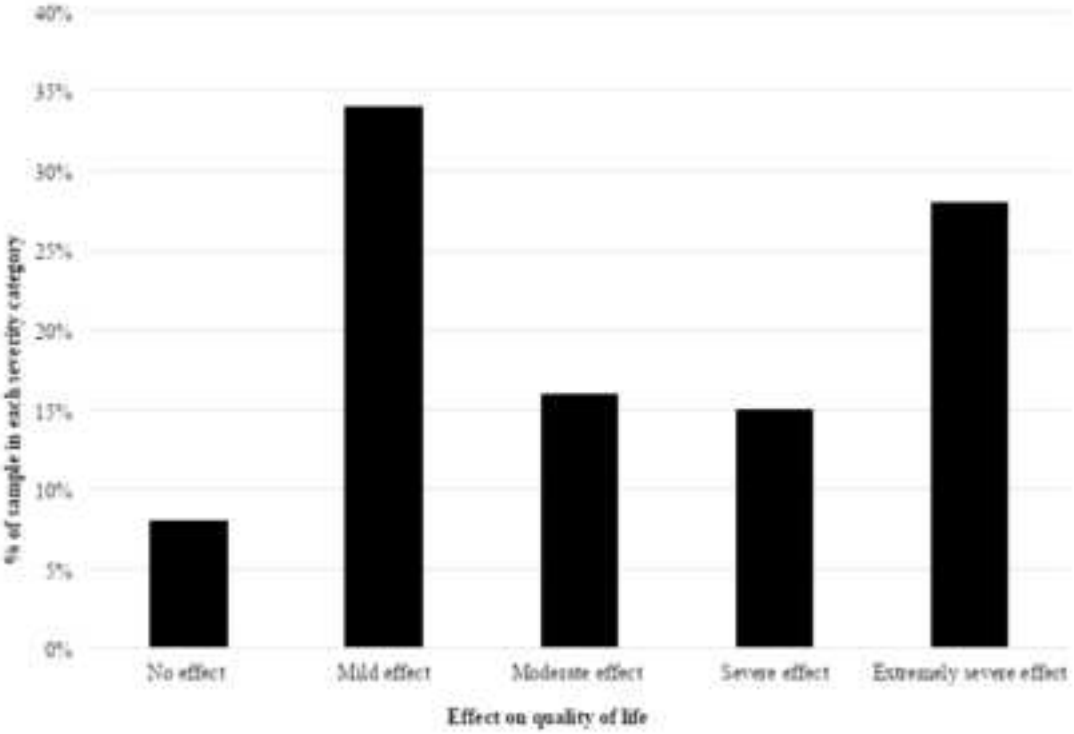

Figure 2. Dermatology quality of life index scores by degrees of severity (n=86)

Point by point response

RE: PNTD-D-23-00994-Quality of life among adults with scabies: a community-based cross-sectional study in north-western Ethiopia

Dear PlosNTDs reviewers and Editors,

Thank you for your continued consideration and the positive review of our manuscript entitled "Quality of life among adults with scabies: a community-based cross-sectional study in north-western Ethiopia".

We have addressed all comments from the three reviewers. The improvements to the manuscript are indicated in tracked changes. Furthermore, the point by point response, in this document, gathered the changes under each comment for easier visualization.

Thank you again.

Yours,

The manuscript co-authors

	**RESPONSE TO REVIEWERS' COMMENTS**
**1**	**Reviewer#1**
1.1.	***Reviewer****: The authors said nothing about their own finding and the context under which this particular work was done. Actually, the Authors summary is to present a concise and non-technical summary of own findings in way understandable for a layperson.*
	**RESPONSE:** As the reviewer rightly pointed out, the author summary lacked focus on the findings of the current study. We have modified to refocus the content. (see tracked changes manuscript lines 65-72).<br><br>'This study presents novel data on severity of quality of life impairment secondary to scabies and which aspects of patients' day-to-day lives are the most affected. This study was conducted in scabies outbreak-affected north-western Ethiopia. Scabies had a moderate impact on the quality of life of people affected. Disease symptoms and their psychosocial implications contributed the most to quality of life impairment. Participants

	of this study came from different sociodemographic backgrounds. Nonetheless, there were no major differences in DLQI scores by background characteristics of the participants.’
1.2.	**Reviewer**: *Line 48-49. The authors claimed that ………. And health education…… is needed to remove “stigma” related to scabies. To my understanding there is not result of theirs that indicated that Scabies actually causes “stigma” and the claim to remove……..perhaps could be due to choice of wording….. in any case such a problem “QoL” or “stigma” could be reduced but complete healing might not be achievable.*<br>**RESPONSE:** We agree that the conclusion feels detached from the main findings of the study. We have made modifications to more clearly show the findings that led to the conclusions. (see tracked changes manuscript lines 48-52).<br><br>Quality of life was moderately impaired among people affected by scabies. Refocusing attention on management of disease symptoms, under the standard scabies treatment, and providing psychosocial support to improve self-perception of people affected with scabies may help reduce quality of life impairment.
1.3.	**Reviewer**: *Line 79-80……. The authors stated that Scabies is amenable to control interventions. Subsequently, presented …..line 98-100…… there was a biannual Ivermectin intervention in the community. But the outbreak is ongoing?? ……. This has two implications for me 1) MDA is a community-based intervention and no sign of abetting the outbreak*<br>**RESPONSE**: Thank you for this comment. The bigger project evaluated the secondary impact of ivermectin based MDA against onchocerciasis on the prevalence of scabies. As the reviewer rightly pointed out, the MDA did not have a secondary impact on scabies epidemiology. However, due to basic differences between MDAs against onchocerciasis and scabies the lack of impact was not completely unexpected. We have published findings of the bigger project elsewhere (doi: 10.1093/trstmh/trad061. PMID: 37665766.).
1.4.	**Reviewer**: *2) the study was conducted under the context when majority of the community members had the disease…..line 290-293….. the authors claimed that such context might have ameliorated the attribute that undermines social functioning! Whereas in line 318…… phrases read as follows…. In a community where more than three quarters prefer*

	avoiding scabies patients……. Related to fear and shame……. To substantiate their finding of high impact in the Symptom and feeling DLQI domain. How could the authors explain own contradictions??<br>**RESPONSE**: We have made changes to indicate that the association between disease epidemiology and stigma was subjective and more of a reduction than complete elimination of stigma. Furthermore, "*In a community where more than three quarters prefer avoiding scabies patients……. Related to fear and shame…….*" this finding was from interviews with scabies-free participants and may not be representative of the rest of the population. We have revised the last sentences of the paragraph to clarify the source of bias to participants. (see tracked changes manuscript lines 338-340).<br><br>'A study among scabies-free participants of the same community reported that more than three quarters of participants preferred to avoid scabies patients (18). Fear and shame were the commonest responses to the anticipation of developing the infestation (18).'
1.5.	**Reviewer:** *Line 294…. Explaining why less effect on "sex and sexuality"……the authors claim absence/less physical or psychosocial impact…… small lesion with limited physical alteration. Whereas, discussing the work and study ….. the phrase reads as ….."the result of inconvenience …..lesions to physical activities. In addition, from line 332…. It reads …. Scabies associated with poverty and hygiene fuels stigma…. Related to fear of transmission …….. limit social interaction…… they claimed though intimate physical contact is implicated for more transmission. Then how is it possible that there claimed "taboo" and the aforementioned explain the context around sex and sexuality.*<br><br>**RESPONSE**: Eight in 10 of our participants were married. We discussed sexual function in light of married people. Due to limited awareness of the mode of transmission, few couples considered sleeping in separate beds, which also likely limited the impact on sexual function. This being the case, we believe this point deserves more explanation and we have added a phrase to the discussion. (see tracked changes manuscript 355-357)<br><br>'[In most Ethiopian cultures, discussing sex is a taboo, more so among married couples (38). In our study, more than 80% of the participants were married. It is likely that most were hesita[illegible] to discuss any change in sexual function with their partners.'

	**Reviewer#2**
2.1.	***Reviewer****: Please see comments on attached pdf*<br>**RESPONSE**: We found all the comments given in the main file very helpful, therefore, we have incorporated nearly all the comments. (see tracked changes in the manuscript)
2.2.	***Reviewer****: I understand that that your study covers most of the areas requested in the STORBE list, at least mention that in the methodology*<br>**RESPONSE**: We have included a sentence indicating that we have followed the STROBE outline (see tracked changes manuscript lines 116-118).<br><br>'Here we summarized aspects of the data collection and analysis that pertain to quality of life by following the STROBE reporting outline. STROBE checklist accompanied this re-submission'
	**Reviewer#3**
3.1.	***Reviewer****: While the introduction provides a definition of quality of life and briefly mentions its relevance in health research, it fails to provide a broader context for the significance of studying quality of life in the context of dermatologic diseases. Readers would benefit from a more detailed discussion of the impact of dermatologic diseases on patients' overall well-being and functioning, as well as the importance of addressing quality of life issues in clinical practice and research*<br>**RESPONSE**: We agree that the introduction would benefit from a more nuanced background about quality of life among people affected by skin diseases. and have expanded on the relevant paragraph. (see tracked changes manuscript lines 80-89).<br><br>'Dermatologic diseases have a serious impact on the quality of life of those affected.. Bodil[illegible] dissatisfaction, anxiety, and depression have been commonly reported among people affect[illegible] with skin diseases (3, 4). Given the chronicity of most dermatologic conditions, the physical and psychosocial impacts are often prolonged (5). Psychosocial impacts result from altered physical appearance, social stigma, and debilitating disease symptoms (6, 7). Severity of impact is determined by the size of lesions, the affected body parts, and symptom duration ([illegible] 8). In previous research, sociodemographic characteristics such as sex, age, income, and marital status were associated with quality of life impairment (5, 7).'

3.2.	***Reviewer**: The introduction briefly mentions that the study aims to assess the effect of scabies on quality of life using the Dermatology Life Quality Index (DLQI) data. However, it could benefit from a clearer and more explicit statement of the research objectives, including specific research questions or hypotheses that the study seeks to address.*<br><br>**RESPONSE**: We have included a sentence describing the objective of the study.<br>(see tracked changes manuscript lines 78-80).<br><br>‘We conducted a population-based cross-sectional study. A survey design was chosen, considering the descriptive nature of the main objective, which was to determine the effect of scabies on the quality of life of patients.’
3.3.	***Reviewer**: The study area section lacks clear organization and structure, making it difficult for readers to follow the study design and data collection procedures. Breaking down the text into subsections such as Study Design, Sampling Procedure, Data Collection, and Data Analysis would enhance readability and comprehension.*<br><br>**RESPONSE**: The methods section is now organized into the recommended subsections.
3.4.	***Reviewer**: The study design is briefly mentioned as a population-based cross-sectional study, however, there is limited detail provided about the rationale behind choosing this design and how it aligns with the research objectives. A clearer explanation of why this design was chosen and how it contributes to answering the research questions is necessary.*<br><br>**RESPONSE**: We have justified why we choose a survey design (see tracked changes manuscript lines 110-112)
3.5.	***Reviewer**: The description of data collection procedures lacks specificity, particularly regarding the training of data collectors and the process of clinical examination. Providing more detail on the training received by data collectors, as well as the methods used for clinical examination and diagnosis of scabies, would enhance the credibility of the study findings.*

	**RESPONSE**: We agree with this important comment. We have included details about the training given to data collectors and also the diagnostic procedure. (see tracked changes manuscript lines 158-163)<br><br>'Five nurses, with ample experience caring for scabies cases, were recruited from the nearby primary hospital to collect data. A dermatovenerologist (WE) from Bahir Dar University ga them a three-day refresher training before data collection. The training was to standardize scabies diagnosis; introduce the data collection tool and create awareness about human subject research ethics.'<br><br>(see tracked changes manuscript lines 164-171)<br><br>'Clinical diagnosis was employed to identify scabies cases. Physical examination of the study participants was conducted inside their house where there was optimal illumination of the examination room. After ensuring privacy, participants were asked to expose body parts that are typically affected by scabietic lesions such as the arms, legs, and the abdomen. However, private body parts were not involved in the examination. Other than physical examination, participants were also asked about contact history with a person presenting wi scabies manifestations.'
	Reviewer*: The discussion could benefit from a section outlining potential future research directions and implications for clinical practice. This could include suggestions for further investigating the relationship between sociodemographic factors and quality of life in scabies patients, exploring interventions to address stigma and psychosocial impacts, and evaluating the effectiveness of integrated care approaches in improving patient outcomes.*<br>**RESPONSE:** Agreeing with this comment we have included a few more research recommendations**.** (see tracked changes manuscript lines 396-400)<br><br>'**…**The sociocultural context forms a critical background to the dynamic relationship between the disease and its effect on quality of life. For this reason, future qualitative studies that can delve deeper into sociocultural factors should be conducted to understand the role of community perception and social norms in mediating the impact of scabies on quality of life of patients.'

	(see tracked changes manuscript lines 409-412)<br><br>‘. . .Though we did not find a statistically significant association, the link between sociodemographic characteristics and quality of life impairment among people with scabies needs further research. We suggest multicentre studies with larger sample sizes to investigate potential differences between the different demographic groups with more accuracy…’

Click here to access/download
Revised Article with Changes Highlighted
Dermatologic life
quality_PlosNTDs_May07_24_Tracked.docx
