# Supplementary material for: Quality of life among adults with scabies: A community-based cross-sectional study in north-western Ethiopia
Source: PLoS Negl Trop Dis. 2024 Aug 20;18(8):e0012429. doi: 10.1371/journal.pntd.0012429 (PMC11364464; doi:10.1371/journal.pntd.0012429)
Supplement: Attachment — Submitted filename: Point by point response_PlosNTDs_Mar01_24.docx [file pntd.0012429.s001.docx]

**Point by point response**

**RE:** PNTD-D-23-00994-Quality of life among adults with scabies: a community-based cross-sectional study in north-western Ethiopia

**Dear PlosNTDs reviewers and Editors,**

Thank you for your continued consideration and the positive review of our manuscript entitled “Quality of life among adults with scabies: a community-based cross-sectional study in north-western Ethiopia”.

We have addressed all comments from all the three reviewers. The improvements to the manuscript are indicated in tracked changes. Furthermore, the point by point response, in this document, gathered the changes under each comment for easier visualization.

Thank you again.

Yours,

The manuscript co-authors

|  | **RESPONSE TO REVIEWERS’ COMMENTS** |
| --- | --- |
| **1** | **Reviewer#1** |
| 1.1. | **Reviewer***: The study context is confusing. line "32" outbreak affected ....since 2015, if the study is conducted in a community where there is uncontrolled outbreak .... this by itself has Psychological implication and bias the DLQI measurement. How could the authors explain this?* |
|  | **RESPONSE:** As the reviewer rightly pointed out, descriptions of the study setting benefit from further explanation to set a clear picture of the context to readers. To this end, we have added a few sentences at the manuscript. (see tracked changes manuscript lines 94-100. Inserted text in [square brackets], deleted text ~~strikethrough~~):  ‘[Scabies prevalence at the time of this study was estimated at 13.4% (19). Despite the high prevalence, there were no community-based scabies control interventions in the study district. Providing standard care at primary health care facilities was the only intervention against the outbreak. Ayu Guagusa was also endemic to onchocerciasis and a bi-annual ivermectin based MDA had been underway since 2015.]’  **RESPONSE:** We agree that the outbreak may affect the DLQI scores. However, scabies is endemic to the study area and the outbreak is believed to date to before 2015, the year when it was first declared. Though we agree with the reviewer and assume the outbreak may limit the effect of scabies on the quality of life of patients, we could not find evidence to support our claim on the existence and direction of effect. We have included sentences describing this in the manuscript (see tracked changes manuscript lines 344-349):  ‘[Apart from presentation, disease pattern may also play a part in determining its effect on quality of life. Scabies had been endemic to north-western Ethiopia and an outbreak was reported in 2015 (18), which was not controlled when this study was conducted. Based on our field observations, we believe the secular trend and large-scale occurrence had contributed to normalizing the disease. This may partly reduce quality of life impairment by ameliorating the attributes that undermine social functioning.]’ |
| 1.2. | **Reviewer***: Study objectives are clearly stated at the end of the introduction, a clear testable hypothesis is not essential on such a study.*  **RESPONSE:** We concur with this comment. The sentence describing the research hypothesis has been removed. |
| 1.3. | **Reviewer**: *In the methods section, the study design is not clearly stated.*  **RESPONSE**: We now have included a few words to further clarify the study design (see tracked changes manuscript lines 101-106).  ‘This [prospective population-based] cross sectional study [used the DLQI tool (16) to determine the impact of scabies on quality of life. It was conducted as] part of a baseline survey for a larger research project that measured the off-target effect of ivermectin-based [mass drug administration (]MDA[)] for onchocerciasis control on scabies prevalence. The [full] design of the [wider] project is detailed elsewhere (17). [Here we summarize the aspects of data collection and analysis specifically related to quality of life.]’ |
| 1.4. | **Reviewer**: *In the study area section: this study occurred during a scabies outbreak? What percentage of the population was affected?*  **RESPONSE**: The study was conducted in scabies outbreak-affected north-western Ethiopia. A related study in the same community estimated a 13.4% prevalence. (see tracked changes manuscript lines 112-118).  ‘[We conducted the study from 5^th^ to 23^rd^ December 2018 in Ayu Guagusa district, north-western Ethiopia, a region that has been affected by scabies outbreak since 2015 (18). Scabies prevalence at the time of this study was estimated at 13.4% (19).]’ |
| 1.5. | **Reviewer:** *Had people already received MDA ivermectin?*  **RESPONSE**: Thank you for this question. We agree that giving a clear account of the scabies control interventions, in the study area, allows readers a better understanding. There had been a bi-annual ivermectin based MDA for onchocerciasis elimination since 2015. However, there was no MDA for scabies control in Ayu Guagusa district. We revised the manuscript to include a few more sentences that help clarify the scabies control strategies at the time of the study. (see tracked changes manuscript lines 112-118)  ‘[Despite the high prevalence, there were no community-based scabies control interventions in the study district. Providing standard care at primary health care facilities was the only intervention against the outbreak. Ayu Guagusa district was also endemic for onchocerciasis. A bi-annual ivermectin based MDA had been underway, since 2015, aiming at onchocerciasis elimination.]’ |
| 1.6. | ***Reviewer****: Is this a very rural community?*  **RESPONSE**: The district comprises 21 predominantly rural *Kebeles* (villages) and all six study *kebeles* were rural. We have revised this section to clarify the study setting (see tracked changes manuscript line 118)  ‘The district comprises 21 predominantly rural *kebeles* (the lowest administrative unit with an average of 500 households) and [and all 6 study kebeles were rural 20]’ |
| 1.7. | ***Reviewer****: A sampling procedure was done to select households in the study area, but then were all household members examined?*  **RESPONSE**: All household members who were available at the time of the survey were examined (see tracked changes manuscript lines 143-148).  ‘[Household heads or adult household members were the informants for household characteristic- related questions. Subsequently, all individual household members who were available at the time of the survey were interviewed and examined for manifestations suggestive of scabies.]’ |
| 1.8. | ***Reviewer****: The sample size was calculated to test a hypothesis? Reference number 20 in this section refers to clinical studies on not studies related to QoL and not sure how reference number 12 is helping in calculating the sample size. Please revise this section carefully.*  **RESPONSE**: The sample size was estimated to obtain an adequate sample to determine the effect of scabies on the quality of life of patients (calculated based on median DLQI score). Reference number 22 details the assumptions for using the formula. The second reference in this section was reference number 13 that is meant to indicate source of the median DLQI score, one of the assumptions used in the sample size calculation (see tracked changes manuscript lines 151-154).  ‘[Sample size was calculated using the formula for a continuous outcome variable in single group studies. The sample size (n=71) was adequate to identify quality of life mean score ($\bar{x}$=10) (13) with a standard deviation of (SD=5.9) and margin of error 1.3]’ |
| 1.9. | ***Reviewer****: Data collection was done by whom? Who diagnosed the patients with scabies? was there a field case-definition used?*  **RESPONSE**: Nurses from a nearby primary hospital conducted the clinical diagnosis and collected sociodemographic data. Clinical diagnosis was used to identify scabies patients. However, no field case definition was used. We made changes to the manuscript to clarify the role of nurses in identifying scabies cases. (see tracked changes manuscript lines 176-178).  ‘[The team collected sociodemographic data and examined people who presented with scabies symptoms by going house-to-house. They examined people of all ages for the parent project, however, in this analysis we used data only from adults.]’ |
| 1.10. | ***Reviewer****: Was there any missing data? How was this handled?*  **RESPONSE**: Thanks for this comment, also made by Reviewer 3. We have revised the manuscript to include details about how missing data was handled (see lines 195-196).  ‘[As the amount of missing data was very small, we have simply reported this descriptively.]’ |
| 1.11. | ***Reviewer:*** *Ref #23 does not explain that sporting activities are rare in this rural study community!*  **RESPONSE**: It is true that the old Ref #23 and the now Ref#25 is an article from a study that validated the DLQI tool for use among podoconiosis patients. In this study impairment to sporting activities contributed the least to the overall effect of podoconiosis on the quality of life of the patients. Our intention was to show that the limited effect on sporting activities resulted from the limited organized sporting activities in rural Ethiopia. We included additional reference to back this claim in the current revised version of the manuscript. (see reference number 27).    ‘[27. Wolde B, Gaudin B. The institutional organization of Ethiopian athletics Annales d'Éthiopie. 2008;XXIII:471-493.]’ |
| 1.12. | **Reviewer**: With regards to treatment was ivermectin MDA given to these participants? Is traditional treatment not tried in this community?  **RESPONSE**: 74.7% of the sample had participated in the last MDA against onchocerciasis, which took place six months before this study. The MDA involved administering ivermectin, a drug that can also cure scabies at the right dose. However, the onchocerciasis MDA involved administering a single dose of ivermectin, which is inadequate to cure scabies. This being the case, we studied all adults who were diagnosed with scabies regardless of their participation in the MDA. However, we did not ask participants whether they used traditional treatment to care for their manifestations. This being the case, the use of traditional treatment is pervasive. Previous studies indicated that nearly 80% of patients used traditional medicine to treat different diseases before seeking care from health facilities. Based on this data we assume a considerable proportion of our study participants did use traditional medicine. |
| 1.13. | **Reviewer**: You need a reference for the adapting the cumulative score and prorating the scores.  **RESPONSE**: We have included a reference. |
| 1.14. | ***Reviewer****: The authors concluded.... timely holistic care.... a bit farfetched and exaggerated. would be good to be explicit what does holistic care entitle give the moderate DLQ implication of Scabies*  **RESPONSE**: Thank you for this important comment. Tailoring the conclusion to specific aspects of symptomatic management and the attributes that contribute to the impairment of psychosocial wellbeing provides a better understanding to readers. We have modified the conclusion along this line (see the revised conclusion lines 50-51).  ‘[Quality of life was moderately impaired among people affected by scabies.  Accompanying scabies treatment with symptomatic management and community based  health education campaigns against stigma associated with scabies may help remove this  impairment.]’ |
| 1.15. | ***Reviewer: 12.*** *Line 24; Scabies undermines ……….. there were only three studies, and reported moderate impact on DLQ…. …. thus is a bit over toned suggest revision.*  **RESPONSE**: We now see the overstatement. We have made changes to give a more reasonable tone to the sentence. (see the revisions lines 93-95).  ‘[Data explaining the effect of scabies on the quality of life impairment secondary to scabies is limited]’ |
| 1.16. | ***Reviewer****: Line 70; complications of scabies….. left unattended……. suggest to be explicit which complications and give references.*  **RESPONSE**: We have modified the sentence in the manuscript specifying which manifestations are important to patients but are not receiving the necessary attention. (see tracked changes manuscript lines 89-91).  ‘[Manifestations of scabies infestation that undermine the quality of life of patients such as skin irritation and itch do not receive the necessary attention]’ |
| 1.17. | ***Reviewer****: Line 17 to 72; lack of evidence …..narrowed case management. If there were known complications which entailed management modifications then what made it narrow? Would these complications also have stake in quality of life of affected individuals.*  **RESPONSE**: We agree that the article would benefit from clarifying this claim. There is an overall evidence gap concerning which aspect of the infestation contributes the most to quality of life impairment. Among these, the effect on psychosocial wellbeing and social functioning of patients is the least studied. More knowledge on these aspects can push scabies management beyond providing cure to the infestation. (see tracked changes manuscript lines 88-91).  ‘[The focus of classic scabies case management is eliminating the mite (7, 11). Often, manifestations of scabies infestation that undermine quality of life of patients such as skin irritation and itch are left unattended. Lack of evidence on scabies and its effect on the quality of life and psychosocial wellbeing of patients partly explains the narrowed areas of focus in scabies case management (12, 13).]’ |
| 1.18. | ***Reviewer****: Line 165; just for curiosity “unemployed” from the context of the study population……. Living on subsistence mixed farming what does it imply.*  **RESPONSE**: The study sample is part of a predominantly rural agrarian community where agriculture is the main economic activity. People referred to as unemployed are those who neither participate in agriculture nor in any other income generating activity. |
| 1.19. | ***Reviewer****: Lines 259-266; the references cited indicated that degree of alteration, body part affected, ……. are associated with degree of DLQ ….. but in your study such variables were not considered; thus could it be arguable that you under/over estimated the implication of scabies as important variables were not included in your study.*  **RESPONSE**: Not measuring the degree of alteration of the skin surface and mapping the most affected body parts is an important limitation of this study. However, the comparison with diseases that cause severe alteration such as podoconiosis and leishmaniasis was meant to signify that extensive alterations to the skin surface predict a more severe quality of life impairment. In a counter argument the relatively mild scabies lesions caused a moderate impairment. (see tracked changes manuscript lines 332-334)  ‘[Comparable to the current findings, studies in China and Brazil reported moderate impairment to quality of life of scabies patients (13, 14). Factors such as degree of alteration to the skin surface; body parts affected; and the type of lesion are important predictors of severity of impairment in people with dermatologic diseases (4, 6).]’ |
| 1.20. | ***Reviewer:*** *Line 273; sexuality: no reference is given to your claim of “taboo”. Also in subsequent paragraphs; from line 299 to 305….. in discussing the implication of scabies in personal relationships …… risky sexual behavior is indicated. In your result it was not seen as such, any reason.*  **RESPONSE**: A reference is cited to back the community’s unfavourable perception of discussing sex.  ‘[Tesso DW, Fantahun MA, Enquselassie F. Parent-young people communication about sexual and reproductive health in E/Wollega zone, West Ethiopia: Implications for interventions. Reproductive Health. 2012;9(1):13.]’  **RESPONSE**: In this study, scabies had a marginal impact on the sexual life of the sample participants. Though anecdotal, the study community perceives scabies more as a disease of poverty and poor hygiene than as a sexually transmitted disease. In the related descriptive study (doi:10.1136/bmjopen-2023-075038.) only 4% of the sample associated scabies with commercial sex workers. |
| 1.21. | ***Reviewer:*** *The exclusion of two items from the standard DLQI scale is mentioned, but the rationale for their exclusion is not discussed in detail. Providing more information on*  *why these items were considered irrelevant could add transparency to the study's methodology.*  **RESPONSE**: We have further elaborated the paragraph to justify the justify the exclusion of the two items from the scale in this study (see tracked changes manuscript lines 161-168).  ‘[However, two of the items were excluded in the current study. Item 6 (sports), due to the rarity of organized sporting activities in this rural study community (26). Item number 10 (treatment) for lack of relevance to population surveys where there is no treatment of cases involved (16). The treatment item was originally deigned to capture the effect of anti-scabietic creams, a source of inconvenience to patients, on quality of life. However, this study was conducted in a community setting and no treatment of patients was involved. Each item had four ordinal response categories with their respective scores (i.e. Not at all=0, a little=1, a lot=2, and very much=3) (16)]’. |
|  | **Reviewer#2** |
| 2.1. | ***Reviewer****: This being a cross-sectional study, have the authors consider completing a STROBE checklist and adding as an appendix?*  **RESPONSE**: We looked up and checked all elements of STROBE checklist are addressed in the manuscript. |
| 2.2. | ***Reviewer****: In the abstract, best to mention how many participants responded (n=86) rather than how many were approached (91)*  **RESPONSE**: Thank you for this comment. Changes have been made to the abstract to report the number of people who participated instead of the number invited to participate (see tracked changes manuscript lines 209-210).  ‘[The study involved 381 households and 86 adults with scabies participated in the study. We used the ten item Cardiff Dermatologic Life Quality Index (DLQI) tool for data collection.]’ |
| 2.3. | ***Reviewer****: The results match the data analysis plan given. Can the authors describe data handling? How was it collected, entered and analysed using which programme?*  **RESPONSE**: Thank you for the valid comment. We have elaborated the data collection procedures. This is a community-based study and data collectors went house to house collecting socio-demographic data and conducting clinical examination on adults who presented with scabies symptoms. We have also described the software package used for the analysis (see tracked changes manuscript lines 176-180).  ‘[The team collected sociodemographic data and examined people who presented with scabies symptoms by going house-to-house. They examined people of all ages for the parent project; however, in this analysis we used data only from adults.  The data were analysed using *Stata 14.0* (StataCorp LLC, Texas) statistical software.]’ |
| 2.4. | ***Reviewer****: Line 178 - correct mis-spelling  "closes".*  **RESPONSE**: Thank you for pointing this out. We have corrected the spelling error. |
| 2.5. | ***Reviewer****: The results are clearly presented, but I have some questions: why was age categorization done this way?*  **RESPONSE**: Existing evidence indicates high scabies burden mong children and adolescents. Categorizing age in short intervals is more informative. However, among adults there is a relatively low prevalence and a uniform distribution across age groups. For this reason, we opted to use data driven categorization. |
| 2.6. | ***Reviewer****: lines 204-205 : better state "we did not observe statistically significant difference..." and maybe comments on some of the differences seen; for example participants in smaller households scored higher.*  **RESPONSE**: Thank you for the comment, we have modified this (see tracked changes manuscript lines 271-274).  ‘[We did not observe a statistically significant difference in ranks of the total DLQI score by sociodemographic characteristics of participants or symptom duration. However, married people and people living in households of the lowest wealth quintile reported higher quality of life impairment than their counterparts.]’ |
| 2.7. | ***Reviewer****: Table 3, the variable "symptom duration" is not described and difficult to understand meaning of result.*  **RESPONSE**: We agree that this could be unclear to readers. We have included a foot note describing “symptom duration”  ‘[^#^ The period between the time of the first symptom and data collection.] |
| 2.8. | ***Reviewer****: Fig 1 , comparing mean of QoL domains this way may not be very clear. Some domains are scored out of 6, and others out of 3. The percentage of domain scores could be calculated from the total possible score for each domain and a comparatively more accurate figure obtained.*  **RESPONSE**: We share the reviewers’ views on this. The domains were calculated based on a varying maximum domain mean score. We agree that changing the mean scores into percentage can contribute to better clarity of the paper. However, the existing cut-off scores to determine degree of severity (Hongbao et.al.,) is calculated based on a cumulative score of all the items in the scale. This limits its application to determine severity of impairment per individual domains. Nonetheless, we have revised the manuscript to further explain how each domain specific mean scores was calculated (see tracked changes manuscript lines 185-187).  ‘[The five domains have a varying maximum possible mean scores. The ‘symptoms and feelings’, ‘daily activity’, and ‘personal relationship’ domains were calculated out of 6 and ‘leisure’ and ‘work and school’ domains out of 3. The difference in ranks of DLQI score by sociodemographic characteristics of participants was determined using Kruskal-Wallis test (26).]’ |
| 2.9. | ***Reviewer****: Fig 2 , 27% of participants  reported that their QoL was  extremely severely affected. This important result is not brought out in your conclusion.*  **RESPONSE**: We have revised the manuscript so that this finding is well reflected in the conclusion.  ‘[The overall effect of scabies on the quality of life of the sample adult scabies patients was moderate and a quarter experienced extremely severe effect. Disease symptoms and self-perception items contributed the most to the overall quality of life impairment. Interventions that aim to improve quality of life, alongside the standard treatment, need to include symptom management.]’ |
| 2.10. | ***Reviewer****: Can the authors comment on how many of the participants diagnosed with scabies and interviewed came from the same household in which others were also affected?*  **RESPONSE**: All scabies cases came from 83 (21.7%) households. From these households 21% contributed 2 or more people affected with scabies to the total sample. |
| 2.11. | ***Reviewer****: This section needs careful revision to relate/ compare the results of this study to findings in other scabies QoL study and QOL in other dermatological conditions in Ethiopia for each paragraph. For example, paragraph starting at line 290 on daily activities: how is this related to the present study participants?*  **RESPONSE:** This section has undergone significant revisions and included literature to enhance comparisons with other NTDs. |
| 2.12. | ***Reviewer****: One important limitation was that the severity of scabies in participant was not recorded or the body parts affected (on line 287 - there is a comments about hands...)*  **RESPONSE**: Existing evidence shows that the hands are one of the most commonly affected parts of the body with scabies lesions. Though in our study we didn’t map body parts affected by the lesion, we assume the bodily distribution of lesions indicated in the literature holds true. For further reference:  <https://link.springer.com/chapter/10.1007/978-3-031-26070-4_15> |
| 2.13. | ***Reviewer****: Line 307 - "male sex is a strong predictor of QoL" - this statement needs clarification*  **RESPONSE**: Thank you for taking note of and pointing out this inconsistency with previous publications. It was a typographical error that we have corrected. We have also added a few more details on the association between sex and quality of life impairment (see tracked changes manuscript lines 408-412).  ‘[A study in Brazil reported that sex plays an important role in shaping the effect of scabies on the quality of life of patients (14). Females were more affected than males and negative self-perception caused by alterations to the skin mediated the impairment (14).]’ |
| 2.14. | ***Reviewer****: The conclusions on lines 311-313 do not seem to be linked to your findings: self -perception and social functioning were the least affected QoL scores in your findings. Which finding reflect the stigmatizing effect of scabies in this group of participants?*  **RESPONSE**: This conclusion is based on the effect of scabies self-perception and social functioning. This is shown in item number 2 in Table 2 and the daily activity domain in figure 1. |
|  | **Reviewer#3** |
| 3.1. | ***Reviewer****: The data analysis section is concise, but it would be beneficial to include more details on how missing data, if any, was handled and whether any statistical software was used. Additionally, specifying the statistical tests used for comparisons would enhance the transparency of the analysis.*  **RESPONSE**: We fully agree with this comment. We have revised the manuscript to include details about how missing data were handled (see lines 195-196).  ‘[As the amount of missing data was very small, we have simply reported this descriptively.]’  **RESPONSE**: We now have described the statistical software (see line 159) and statistical tests used in comparing the data (see lines 180-181).  ‘[The data were analysed using *Stata 14.0* (StataCorp LLC, Texas) statistical software.]’  ‘[The total and DLQ domain specific mean scores (mDLQI) and standard deviations were calculated. The difference in ranks of DLQI score by sociodemographic characteristics of participants was determined using the Kruskal-Wallis test (27).]’ |
| 3.2. | ***Reviewer****: The discussion could benefit from a more explicit comparison with similar studies conducted in other regions or countries. This would provide a broader context for understanding whether the impact on quality of life in northern Ethiopia is consistent with or differs from findings in diverse cultural and geographical settings.*  **RESPONSE**: We agree with the reviewer. However, literature on the effect of scabies on quality of life of patients is limited. In an extensive literature search, we could only find two articles on the subject and both are referenced in the discussion. This has limited our ability to compare our findings with other studies. Nonetheless, following this comment by the reviewer we have tried to expand the discussion by making comparisons with literature on other NTDs in Ethiopia. |
| 3.3. | ***Reviewer****: The discussion mentions that sociodemographic characteristics were not associated with quality of life impairment. However, it could be valuable to elaborate on why certain sociodemographic factors were expected to have an impact and explore potential reasons for the lack of association in this specific context.*  **RESPONSE**: The lack of association between sociodemographic characteristics and quality of life impairment is further elaborated in line with your comments (please see line 408-415).  ‘[Sociodemographic characteristics and duration of disease symptoms were not associated  with quality of life impairment. In another study sex shaped the effect of scabies on the  quality of life of patients (14). Females were more affected than males and negative self-  perception, caused by alterations to the skin, mediated the impairment (14). Contrary to this,  the current study was conducted in a traditional community where dresses of women  typically cover most parts of their body. This can limit the severity of impairment to quality of  life by minimizing the effect on a range of behaviour including clothing preferences; daily  activity; and self-perception.]’ |
| 3.4. | ***Reviewer****: The discussion mentions stigma and social isolation as significant contributors to quality of life impairment. Providing more in-depth insights into the nature and extent of stigma, as well as potential interventions to address it, would contribute to a more nuanced understanding.*  **RESPONSE**: This is a valid comment. The scope of stigma and the necessary interventions to avert its impact on the well-being of scabies patients is further explained in the revised manuscript (see tracked changes manuscript lines 379-383 & 393-402).  ‘[In the study community, where more than three quarters prefer to avoiding scabies patients (18), fear and shame are the commonest responses to the anticipation of developing the infestation (18). This pervasive stigma can have a toll on the mental health of scabies patients warranting psychosocial interventions alongside clinical care.]’  ‘[In many communities, scabies is associated with poverty and lack of hygiene (44, 50). Such misconceptions can fuel social stigma against scabies patients. A related literature in this same community reported that 37% of the sample preferred to avoid scabies patients (18). These circumstances can lead to patients limiting social relations in fear of rejection. The other reason for limiting social interactions can be to prevent transmission. Patients who are aware of the increased risk of transmission with increasing physical proximity may isolate themselves to protect people who are close to them (50).]’ |
| 3.5. | ***Reviewer****: While the sampling procedure is mentioned, it lacks a detailed explanation of how the random selection of households was conducted. Providing a step-by-step description or referencing the specific publication where it is explained would be beneficial for transparency and reproducibility.*  **RESPONSE**: Thank you for this input. We have included a reference, from the larger project, that details the sampling procedure step-by-step (See reference number 17 in the bibliography).  ‘[Yirgu R, Middleton J, Fekadu A, Cassell JA, Tesfaye A, Jones CI, et al. Scabies in the Amhara region of northern Ethiopia: a cross-sectional study of prevalence, determinants, clinical presentation, and community knowledge. BMJ Open. 2023;13(e075038).]’ |
