# Supplementary material for: Quality of life among adults with scabies: A community-based cross-sectional study in north-western Ethiopia
Source: PLoS Negl Trop Dis. 2024 Aug 20;18(8):e0012429. doi: 10.1371/journal.pntd.0012429 (PMC11364464; doi:10.1371/journal.pntd.0012429)
Supplement: Attachment — Submitted filename: Point by point response_PlosNTDs_May07_24 GD SB.docx [file pntd.0012429.s003.docx]

**Point by point response**

**RE:** PNTD-D-23-00994-Quality of life among adults with scabies: a community-based cross-sectional study in north-western Ethiopia

**Dear PlosNTDs reviewers and Editors,**

Thank you for your continued consideration and the positive review of our manuscript entitled “Quality of life among adults with scabies: a community-based cross-sectional study in north-western Ethiopia”.

We have addressed all comments from the three reviewers. The improvements to the manuscript are indicated in tracked changes. Furthermore, the point by point response, in this document, gathered the changes under each comment for easier visualization.

Thank you again.

Yours,

The manuscript co-authors

|  | **RESPONSE TO REVIEWERS’ COMMENTS** |
| --- | --- |
| **1** | **Reviewer#1** |
| 1.1. | ***Reviewer****: The authors said nothing about their own finding and the context under which this particular work was done. Actually, the Authors summary is to present a concise and non-technical summary of own findings in way understandable for a layperson.* |
|  | **RESPONSE:** As the reviewer rightly pointed out, the author summary lacked focus on the findings of the current study. We have modified to refocus the content. (see tracked changes manuscript lines 65-72).  ‘This study presents novel data on severity of quality of life impairment secondary to scabies and which aspects of patients’ day-to-day lives are the most affected. This study was conducted in scabies outbreak-affected north-western Ethiopia. Scabies had a moderate impact on the quality of life of people affected. Disease symptoms and their psychosocial implications contributed the most to quality of life impairment. Participants of this study came from different sociodemographic backgrounds. Nonetheless, there were no major differences in DLQI scores by background characteristics of the participants.’ |
| 1.2. | **Reviewer***: Line 48-49. The authors claimed that ………. And health education…… is needed to remove “stigma” related to scabies. To my understanding there is not result of theirs that indicated that Scabies actually causes “stigma” and the claim to remove……..perhaps could be due to choice of wording….. in any case such a problem “QoL” or “stigma” could be reduced but complete healing might not be achievable.*  **RESPONSE:** We agree that the conclusion feels detached from the main findings of the study. We have made modifications to more clearly show the findings that led to the conclusions. (see tracked changes manuscript lines 48-52).  Quality of life was moderately impaired among people affected by scabies. Refocusing  attention on management of disease symptoms, under the standard scabies treatment, and  providing psychosocial support to improve self-perception of people affected with  scabies may help reduce quality of life impairment. |
| 1.3. | **Reviewer**: *Line 79-80……. The authors stated that Scabies is amenable to control interventions. Subsequently, presented …..line 98-100…… there was a biannual Ivermectin intervention in the community. But the outbreak is ongoing?? ……. This has two implications for me 1) MDA is a community-based intervention and no sign of abetting the outbreak*  **RESPONSE**: Thank you for this comment. The bigger project evaluated the secondary impact of ivermectin based MDA against onchocerciasis on the prevalence of scabies. As the reviewer rightly pointed out, the MDA did not have a secondary impact on scabies epidemiology. However, due to basic differences between MDAs against onchocerciasis and scabies the lack of impact was not completely unexpected. We have published findings of the bigger project elsewhere (doi: 10.1093/trstmh/trad061. PMID: 37665766.). |
| 1.4. | **Reviewer**: *2) the study was conducted under the context when majority of the community members had the disease…..line 290-293….. the authors claimed that such context might have ameliorated the attribute that undermines social functioning! Whereas in line 318…… phrases read as follows…. In a community where more than three quarters prefer avoiding scabies patients……. Related to fear and shame……. To substantiate their finding of high impact in the Symptom and feeling DLQI domain. How could the authors explain own contradictions??*  **RESPONSE**: We have made changes to indicate that the association between disease epidemiology and stigma was subjective and more of a reduction than complete elimination of stigma. Furthermore, “*In a community where more than three quarters prefer avoiding scabies patients……. Related to fear and shame…….”* this finding was from interviews with scabies-free participants and may not be representative of the rest of the population. We have revised the last sentences of the paragraph to clarify the source of bias to participants. (see tracked changes manuscript lines 338-340).  ‘A study among scabies-free participants of the same community reported that more than three quarters of participants preferred to avoid scabies patients (18). Fear and shame were the commonest responses to the anticipation of developing the infestation (18).’ |
| 1.5. | **Reviewer:** *Line 294…. Explaining why less effect on “sex and sexuality”……the authors claim absence/less physical or psychosocial impact…… small lesion with limited physical alteration. Whereas, discussing the work and study ….. the phrase reads as …..”the result of inconvenience …..lesions to physical activities. In addition, from line 332…. It reads …. Scabies associated with poverty and hygiene fuels stigma…. Related to fear of transmission …….. limit social interaction…… they claimed though intimate physical contact is implicated for more transmission. Then how is it possible that there claimed “taboo” and the aforementioned explain the context around sex and sexuality.*  **RESPONSE**: Eight in 10 of our participants were married. We discussed sexual function in light of married people. Due to limited awareness of the mode of transmission, few couples considered sleeping in separate beds, which also likely limited the impact on sexual function. This being the case, we believe this point deserves more explanation and we have added a phrase to the discussion. (see tracked changes manuscript 355-357)  ‘[In most Ethiopian cultures, discussing sex is a taboo, more so among married couples (38). In our study, more than 80% of the participants were married. It is likely that most were hesitant to discuss any change in sexual function with their partners.’ |
|  | **Reviewer#2** |
| 2.1. | ***Reviewer****: Please see comments on attached pdf*  **RESPONSE**: We found all the comments given in the main file very helpful, therefore, we have incorporated nearly all the comments. (see tracked changes in the manuscript) |
| 2.2. | ***Reviewer****: I understand that that your study covers most of the areas requested in the STORBE list, at least mention that in the methodology*  **RESPONSE**: We have included a sentence indicating that we have followed the STROBE outline (see tracked changes manuscript lines 116-118).  ‘Here we summarized aspects of the data collection and analysis that pertain to quality of life by following the STROBE reporting outline. STROBE checklist accompanied this re-submission’ |
|  | **Reviewer#3** |
| 3.1. | ***Reviewer****: While the introduction provides a definition of quality of life and briefly mentions its relevance in health research, it fails to provide a broader context for the significance of studying quality of life in the context of dermatologic diseases. Readers would benefit from a more detailed discussion of the impact of dermatologic diseases on patients' overall well-being and functioning, as well as the importance of addressing quality of life issues in clinical practice and research*  **RESPONSE**: We agree that the introduction would benefit from a more nuanced background about quality of life among people affected by skin diseases. and have expanded on the relevant paragraph. (see tracked changes manuscript lines 80-89).  ‘Dermatologic diseases have a serious impact on the quality of life of those affected.. Bodily dissatisfaction, anxiety, and depression have been commonly reported among people affected with skin diseases (3, 4). Given the chronicity of most dermatologic conditions, the physical and psychosocial impacts are often prolonged (5). Psychosocial impacts result from altered physical appearance, social stigma, and debilitating disease symptoms (6, 7). Severity of impact is determined by the size of lesions, the affected body parts, and symptom duration (5, 8). In previous research, sociodemographic characteristics such as sex, age, income, and marital status were associated with quality of life impairment (5, 7).’ |
| 3.2. | ***Reviewer****: The introduction briefly mentions that the study aims to assess the effect of scabies on quality of life using the Dermatology Life Quality Index (DLQI) data. However, it could benefit from a clearer and more explicit statement of the research objectives, including specific research questions or hypotheses that the study seeks to address.*  **RESPONSE**: We have included a sentence describing the objective of the study. (see tracked changes manuscript lines 78-80).  ‘We conducted a population-based cross-sectional study. A survey design was chosen, considering the descriptive nature of the main objective, which was to determine the effect of scabies on the quality of life of patients.’ |
| 3.3. | ***Reviewer****: The study area section lacks clear organization and structure, making it difficult for readers to follow the study design and data collection procedures. Breaking down the text into subsections such as Study Design, Sampling Procedure, Data Collection, and Data Analysis would enhance readability and comprehension.*  **RESPONSE**: The methods section is now organized into the recommended subsections. |
| 3.4. | ***Reviewer****: The study design is briefly mentioned as a population-based cross-sectional study, however, there is limited detail provided about the rationale behind choosing this design and how it aligns with the research objectives. A clearer explanation of why this design was chosen and how it contributes to answering the research questions is necessary.*  **RESPONSE**: We have justified why we choose a survey design (see tracked changes manuscript lines 110-112) |
| 3.5. | ***Reviewer****: The description of data collection procedures lacks specificity, particularly regarding the training of data collectors and the process of clinical examination. Providing more detail on the training received by data collectors, as well as the methods used for clinical examination and diagnosis of scabies, would enhance the credibility of the study findings.*  **RESPONSE**: We agree with this important comment. We have included details about the training given to data collectors and also the diagnostic procedure. (see tracked changes manuscript lines 158-163)  ‘Five nurses, with ample experience caring for scabies cases, were recruited from the nearby primary hospital to collect data. A dermatovenerologist (WE) from Bahir Dar University gave them a three-day refresher training before data collection. The training was to standardize scabies diagnosis; introduce the data collection tool and create awareness about human subject research ethics.’  (see tracked changes manuscript lines 164-171)  ‘Clinical diagnosis was employed to identify scabies cases. Physical examination of the  study participants was conducted inside their house where there was optimal illumination of the examination room. After ensuring privacy, participants were asked to expose body parts that are typically affected by scabietic lesions such as the arms, legs, and the abdomen. However, private body parts were not involved in the examination. Other than physical examination, participants were also asked about contact history with a person presenting with scabies manifestations.’ |
|  | ***Reviewer****: The discussion could benefit from a section outlining potential future research directions and implications for clinical practice. This could include suggestions for further investigating the relationship between sociodemographic factors and quality of life in scabies patients, exploring interventions to address stigma and psychosocial impacts, and evaluating the effectiveness of integrated care approaches in improving patient outcomes.*  **RESPONSE:** Agreeing with this comment we have included a few more research recommendations**.** (see tracked changes manuscript lines 396-400)  **‘…**The sociocultural context forms a critical background to the dynamic relationship between the disease and its effect on quality of life. For this reason, future qualitative studies that can delve deeper into sociocultural factors should be conducted to understand the role of community perception and social norms in mediating the impact of scabies on quality of life of patients.’  (see tracked changes manuscript lines 409-412)  ‘. . .Though we did not find a statistically significant association, the link between sociodemographic characteristics and quality of life impairment among people with scabies needs further research. We suggest multicentre studies with larger sample sizes to investigate potential differences between the different demographic groups with more accuracy…’ |
