# Supplementary material for: Quality of life among adults with scabies: A community-based cross-sectional study in north-western Ethiopia
Source: PLoS Negl Trop Dis. 2024 Aug 20;18(8):e0012429. doi: 10.1371/journal.pntd.0012429 (PMC11364464; doi:10.1371/journal.pntd.0012429)
Supplement: Attachment — Submitted filename: Cover letter_PlosNTDs_July06_24.docx [file pntd.0012429.s005.docx]

15/07/2024

**Point by point response**

**RE:** PNTD-D-23-00994-Quality of life among adults with scabies: a community-based cross-sectional study in north-western Ethiopia

**Dear PlosNTDs reviewers and Editors,**

Thank you for your continued consideration and the positive review of our manuscript entitled “Quality of life among adults with scabies: a community-based cross-sectional study in north-western Ethiopia”.

The only review feedback in this round was to improve the language. We have corrected grammatical and typographical errors as indicated in tracked changes.

Thank you
